# Dosage Sensitivity of RPL9 and Concerted Evolution of Ribosomal Protein Genes in Plants

**DOI:** 10.3389/fpls.2015.01102

**Published:** 2015-12-16

**Authors:** Deborah Devis, Sue M. Firth, Zhe Liang, Mary E. Byrne

**Affiliations:** School of Biological Sciences, The University of Sydney, SydneyNSW, Australia

**Keywords:** concerted evolution, dosage sensitive, gene redundancy, plant, ribosomal protein

## Abstract

The ribosome in higher eukaryotes is a large macromolecular complex composed of four rRNAs and eighty different ribosomal proteins. In plants, each ribosomal protein is encoded by multiple genes. Duplicate genes within a family are often necessary to provide a threshold dose of a ribosomal protein but in some instances appear to have non-redundant functions. Here, we addressed whether divergent members of the *RPL9* gene family are dosage sensitive or whether these genes have non-overlapping functions. The *RPL9* family in *Arabidopsis thaliana* comprises two nearly identical members, *RPL9B* and *RPL9C*, and a more divergent member, *RPL9D*. Mutations in *RPL9C* and *RPL9D* genes lead to delayed growth early in development, and loss of both genes is embryo lethal, indicating that these are dosage-sensitive and redundant genes. Phylogenetic analysis of *RPL9* as well as *RPL4*, *RPL5*, *RPL27a*, *RPL36a*, and *RPS6* family genes in the Brassicaceae indicated that multicopy ribosomal protein genes have been largely retained following whole genome duplication. However, these gene families also show instances of tandem duplication, small scale deletion, and evidence of gene conversion. Furthermore, phylogenetic analysis of *RPL9* genes in angiosperm species showed that genes within a species are more closely related to each other than to *RPL9* genes in other species, suggesting ribosomal protein genes undergo convergent evolution. Our analysis indicates that ribosomal protein gene retention following whole genome duplication contributes to the number of genes in a family. However, small scale rearrangements influence copy number and likely drive concerted evolution of these dosage-sensitive genes.

## Introduction

The 80S ribosome of higher eukaryotes is a macromolecular complex composed of two subunits, a large 60S subunit and a small 40S subunit. The 60S subunit comprises 28S, 5.8S, and 5S rRNA and 47 ribosomal proteins. The 40S subunit is composed of 18S rRNA and 33 proteins (Melnikov et al., 2012). Ribosomes are produced through a cascade of events involving coordinated processing of precursor rRNA, progressive association of individual ribosomal proteins with rRNA, export of pre-ribosome particles from the nucleolus to the cytoplasm, and assembly to form mature subunits. The two subunits join at translation initiation to form a ribosome, which carries out protein synthesis.

In *Arabidopsis thaliana*, each ribosomal protein is encoded by multiple genes (Barakat et al., 2001). Duplicate ribosomal protein genes may serve to provide a critical dose of a ribosomal protein or may provide distinct functions through differential expression or through diversification of protein function (Horiguchi et al., 2012; Xue and Barna, 2012). In *Arabidopsis*, mutations in members of a ribosomal protein family may have different phenotypic outcomes but more often show similar dose-dependent phenotypes (Degenhardt and Bonham-Smith, 2008; Yao et al., 2008; Fujikura et al., 2009; Creff et al., 2010; Rosado and Raikhel, 2010; Rosado et al., 2010; Horiguchi et al., 2011; Stirnberg et al., 2012; Casanova-Sáez et al., 2014; Zsögön et al., 2014). Mutations in *A. thaliana* ribosomal protein genes are generally recessive, and only two semi-dominant mutants have been described (Byrne, 2009; Horiguchi et al., 2012). *Arabidopsis Minute-like1* (*aml1*) has a mutation in the *RPS5B* gene and homozygous mutants arrest during early stages of embryo development. Hemizygous plants are viable and have a range of phenotypes including reduced seedling size and altered organ vascular patterning (Weijers et al., 2001). *rpl27ac-1d* is a dominant-negative mutation in the *RPL27aC* gene and homozygous plants have abnormal development of embryos and pleiotropic defects in the plant shoot. Heterozygous plants are slow growing with distinct developmental phenotypes, including pointed and serrated leaves (Szakonyi and Byrne, 2011a,b). Increasing the ratio of *rpl27ac-1d* relative to wild type results in a progressive increase in the range and severity of phenotypes consistent with plant growth and development being sensitive to the dose of RPL27a (Zsögön et al., 2014).

RPL27a is encoded by two redundant genes. Loss-of-function mutations in *RPL27aC* and *RPL27aB* have mild and no leaf phenotype, respectively, whereas double heterozygote plants have a pointed and serrated leaf shape phenotype (Zsögön et al., 2014). Mutations in both *RPL27aB* and *RPL27aC* genes are not transmitted through gametes indicating dramatically reduced levels of RPL27a is haploid lethal (Zsögön et al., 2014). Likewise ribosomal proteins RPL4, RPL5, RPL36a, and RPS6, are each encoded by two functional genes. For each of these duplicate genes, single mutants are viable and plants display a pointed and serrated leaf phenotype that is characteristic of mutations in *A. thaliana* ribosomal protein genes. Double heterozygous mutants for both genes within a family also display these leaf phenotypes and mutant alleles in duplicate genes are not transmitted through gametes (Yao et al., 2008; Fujikura et al., 2009; Creff et al., 2010; Rosado et al., 2010; Casanova-Sáez et al., 2014). These phenotypes indicate that members of these ribosomal protein families are redundant and that the duplicate genes in a family are required for production of sufficient levels of a ribosomal protein for viability of haploid gametes and for plant growth.

Duplicate genes may arise through whole or partial genome duplication, or through tandem gene duplication. Many flowering plants are ancient polyploids and retain evidence of past genome duplications (Van de Peer et al., 2009). Duplicate genes created through genome duplication either diverge in function or one duplicate is lost from the genome. However, gene loss is biased and dosage sensitive genes appear to be preferentially retained following genome duplication. According to the gene balance hypothesis, following whole genome duplication, an unfavorable imbalance in the optimum ratio of proteins may arise from loss of genes that code for components of a protein complex or components in a molecular pathway. As such dosage-sensitive genes may be retained following whole genome duplication in order to maintain a balance in the concentration of proteins in complex or in a molecular pathway (Veitia, 2002; Papp et al., 2003; Birchler and Veitia, 2012). Consistent with the gene balance hypothesis, multiple plant species display evidence of over-retention of genes within the ontology category of “ribosome” following genome duplication (Blanc and Wolfe, 2004; Maere et al., 2005; Rizzon et al., 2006; Thomas et al., 2006; Wang et al., 2011; Jiang et al., 2013).

Although there is an overall trend toward retention of ribosomal protein genes post-genome duplication, there has been limited analysis of the evolution of specific cytoplasmic ribosomal protein gene families within plants. Here we demonstrate that two divergent members of the ribosomal protein family *RPL9*, *RPL9C*, and *RPL9D*, are dosage-sensitive and redundant, indicating that these duplicate ribosomal protein genes serve to maintain adequate levels of a ribosomal protein for sufficient ribosome production. Analysis of *RPL9* family genes in Brassicaceae species, and more broadly within eudicots and monocots revealed limited *RPL9* copy number variation between species. In the Brassicaceae, *RPL9* copy number appears to be the outcome of multiple genome rearrangements including whole genome duplication, tandem duplication and gene loss. Furthermore nucleotide sequence variation between *RPL9* genes within a species appears to be driven toward homogenization, likely through gene conversion. Analysis of *RPL4*, *RPL5*, *RPL27a*, *RPL36a*, and *RPS6* genes in the Brassicaceae reveals dynamic evolution of ribosomal protein gene families.

## Materials and Methods

### Plant Materials and Growth Conditions

*Arabidopsis* mutant *rpl9c-1* (formerly published as *piggyback2-1* (*pgy2-1*)) has been described previously (Pinon et al., 2008). *rpl9d-1* (SALK_111804) was obtained from The European *Arabidopsis* Stock Center (Scholl et al., 2000) and was backcrossed five times to Landsberg *erecta* prior to genetic analysis. Plants were grown in soil at 22°C with a day length of 16 h. Growth measurement data from eight plants of each genotype were analyzed using SPSS Statistics for Macintosh, Version 22.0 (IBM Corporation). One-way or repeated measures analysis of variance (ANOVA) tests were performed, followed by Scheffe’s multiple comparison *post hoc* test and *P* < 0.05 were considered as significant.

### Molecular Biology

The genotype of wild type and mutant *rpl9c* and *rpl9d* alleles was determined by PCR using gene specific primers. *RPL9D:RPL9D* was generated by PCR amplification of an 4.5 kb genomic region encompassing *RPL9D* and cloning into the binary vector pMDC123 (Curtis and Grossniklaus, 2003). The construct was transformed into *rpl9c* by floral dip (Clough and Bent, 1998).

### Phylogenetic and Synteny Analysis

Gene sequences were obtained from Phytozome (Goodstein et al., 2012). Designated gene names used in phylogenetic analysis and corresponding genomic unique locus identifiers are listed in Supplementary Tables S1–S8. Brassicaceae species included *A. thaliana*, *Arabidopsis lyrata*, *Capsella rubella*, and *Capsella grandiflora*, within the Camelineae, and *Eutrema salsugineum* (formerly *Thellungiella halophila*) and *B. rapa*. For Brassicaceae species, genome sequence assembly into chromosomes was incomplete for several species. Therefore designated *RPL9, RPL4*, *RPL5*, *RPL27a*, *RPL36a*, and *RPS6* gene names within a species were based on phylogenetic relationships. *RPL9* gene names for other dicot species and for monocot species that had complete genome assemblies were assigned according to map location. This included the dicot species *Gossypium raimondii, Medicago truncatula, Phaseolus vulgaris, Poplar trichocarpa, Solanum lycopersicum*, *S. tuberosum*, and *Vitis vinifera*, and the monocot species *Brachypodium distachyon, Oryza sativa, Sorghum bicolor*, and *Zea mays. RPL9* gene names for species where genome assembly was incomplete were arbitrarily assigned. This included the dicot species *Aquilegia coerulea, Carica papaya, Citrus clementina, Citrus sinensis, Linum usitatissimum*, and *Mimulus guttatus*, and the monocot species *Panicum virgatum* and *Setaria italica*. The CDS sequences were used to estimate phylogenetic relationships. Orthologous ribosomal proteins from *Drosophila melanogaster* were selected as the outgroup. Full-length sequences were aligned using ClustalW and phylogenetic relationships were inferred using MEGA6.06 (Tamura et al., 2013). Trees were constructed with the Maximum-Likelihood algorithm and default settings with 1000 bootstrap replications. Synteny analysis was carried out using CoGepedia (Lyons and Freeling, 2008; Lyons et al., 2008) with a sequence distance set to 100 kb.

## Results

### *RPL9C* and *RPL9D* have Redundant Functions in Plant Growth

**FIGURE 1:**
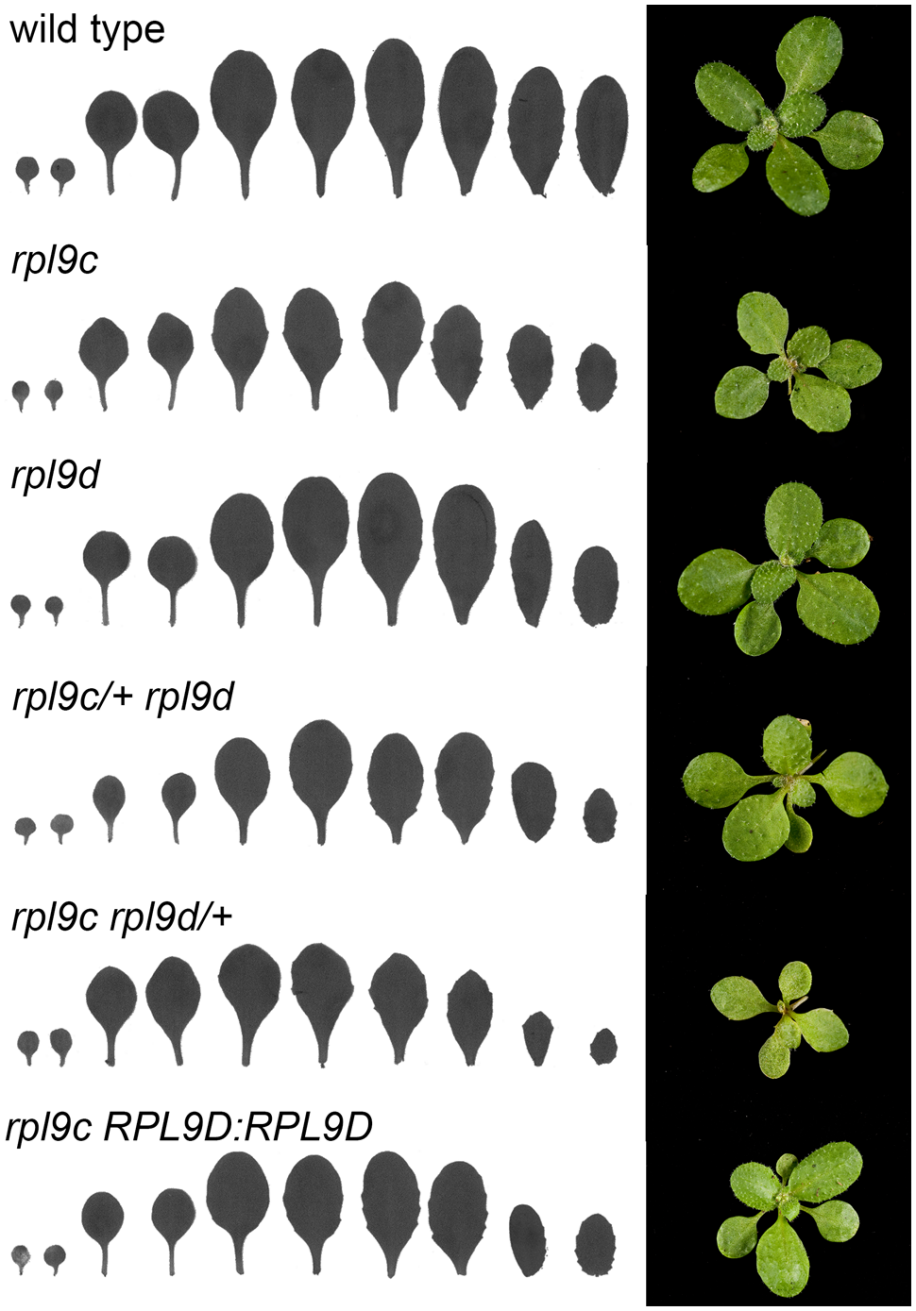
***RPL9C* and *RPL9D* have redundant functions in leaf development.** Silhouettes **(left)** and rosettes **(right)** of wild type, *rpl9c*, *rpl9d, rpl9c/+ rpl9d, rpl9c rpl9d/+*, and *rpl9c* homozygous for the transgene *RPL9D:RPL9D*.

*Arabidopsis thaliana* has three *RPL9* genes, *RPL9B*, *RPL9C*, and *RPL9D* (Barakat et al., 2001). The proteins encoded by *RPL9B* and *RPL9C* share 100% amino acid identity whereas *RPL9B/RPL9C* and *RPL9D* encoded proteins are more divergent and share 89% amino acid identity. Although all three genes are ubiquitously expressed, transcript levels of *RPL9C* are approximately twofold higher than *RPL9D* and threefold higher than *RPL9B* (Laubinger et al., 2008; Pinon et al., 2008). To determine whether divergent members of the *RPL9* gene family are redundant we compared phenotypes resulting from mutation in *RPL9C* and *RPL9D*. *rpl9c* (previously named *pgy2*) is a weak allele and has a splice-donor site point mutation that reduces the level of wild type transcript. *rpl9c* leaves are pointed and have more prominent marginal serrations compared to wild type (Pinon et al., 2008) (**Figure 1**). A T-DNA mutant *rpl9d* had an insertion in the first exon of *RPL9D* and was predicted to be a null allele. The leaf shape of *rpl9d* was not distinct from that of wild type (**Figure 1**). This indicated either *RPL9D* has no function in leaf development or the contribution of *RPL9D* to leaf development is not significant in the presence of functional *RPL9B* and *RPL9C*. To determine whether or not *RPL9D* contributes to leaf development, the effect of reduced levels of *RPL9C* in the *rpl9d* mutant was examined. The double homozygous mutant is embryo lethal (see below). Therefore *rpl9c/+ rpl9d* plants were examined. *rpl9c/+ rpl9d* plants had mildly serrated leaves compared with *rpl9d* single mutants indicating partial loss of *RPL9C* function slightly modifies leaf development in the *rpl9d* background (**Figure 1**). The effect of reduced *RPL9D* on the *rpl9c* mutant was also examined. The leaf phenotype of *rpl9c rpl9d/+* plants was more severe and leaves were smaller and more pointed than *rpl9c* (**Figure 1**). This enhanced phenotype indicates *RPL9D* acts redundantly with *RPL9C* in leaf growth.

To confirm that *RPL9D* is redundant with *RPL9C*, a genomic clone *RPL9D:RPL9D*, encompassing the gene promoter and coding region, was transformed into the *rpl9c* mutant to test for complementation. The leaf shape of progeny from 10 independent transformants was examined. All ten lines segregated plants that had a wild-type phenotype. Progeny from phenotypically wild type plants in two lines were confirmed as homozygous for *RPL9D:RPL9D* (**Figure 1**). *RPL9D:RPL9D* is therefore able to replace the function of *RPL9C*.

**FIGURE 2:**
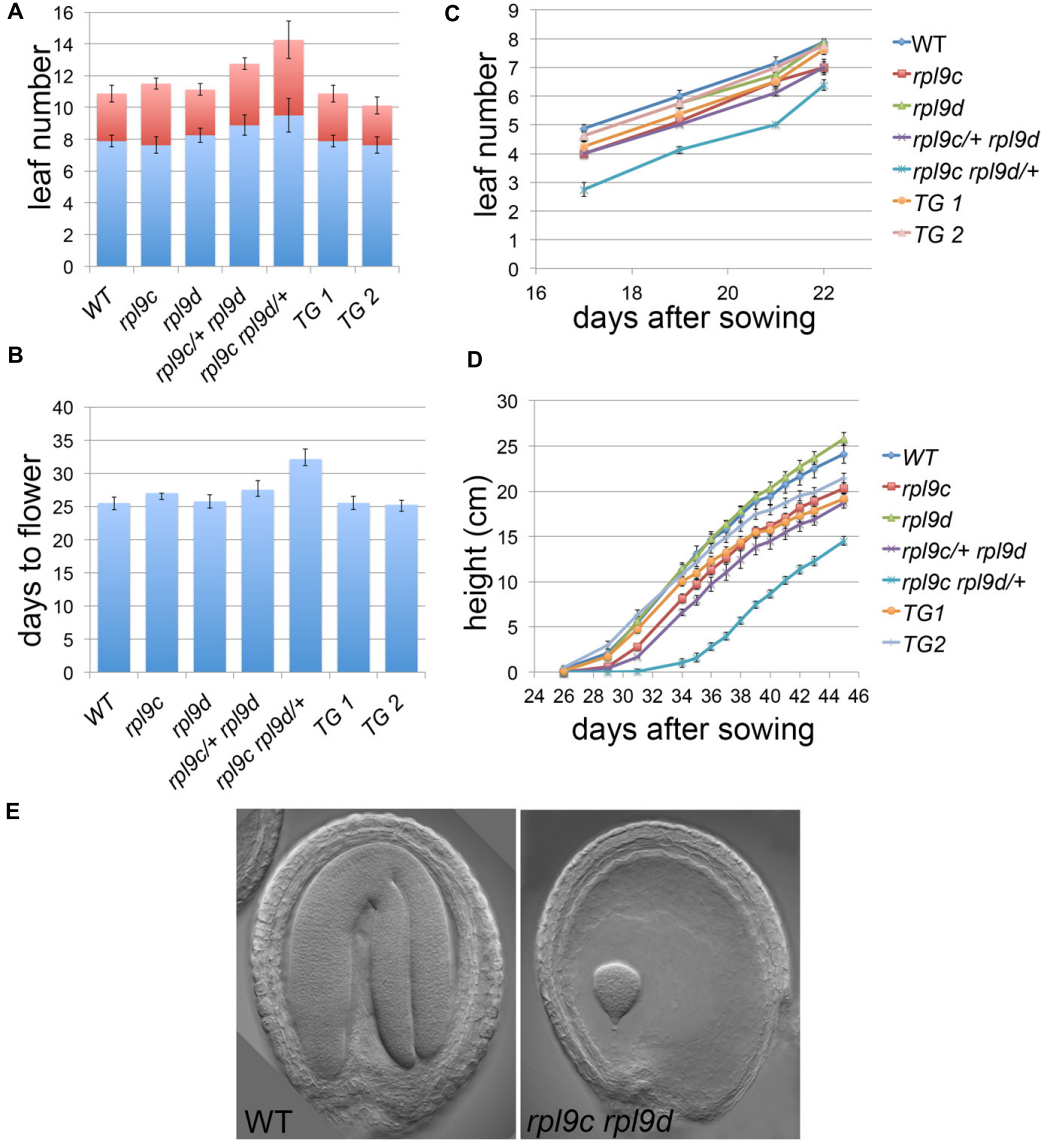
**Mutations in *RPL9* genes delay growth.** The number of rosette (blue) and cauline (red) leaves **(A)**, days to flower **(B)**, rate of rosette leaf emergence **(C)**, and rate of inflorescence growth **(D)** are shown for the genotypes wild type, *rpl9c*, *rpl9d, rpl9c/+ rpl9d, rpl9c rpl9d/+*, and for two independent lines *rpl9c* homozygous for the transgene *RPL9D:RPL9D* (TG1 and TG2). Data is the mean ± SE (*n* = 8). Embryos from a silique of an *rpl9c/+ rpl9d* plant **(E)**. A mature wild type embryo (left) and a putative homozygous *rpl9c rpl9d* embryo arrested at a late globular stage of development (right).

In addition to leaf shape, mutations in ribosomal proteins result in slow growth, although there is limited information quantifying this growth defect. To compare the rate of growth of *rpl9* mutants relative to wild type, we measured the rate of leaf production, the time to flower and the rate of inflorescence elongation for wild type, *rpl9c, rpl9d, rpl9c/+ rpl9d*, and *rpl9c rpl9d/+* plants. Plants from two independent *rpl9c RPL9D:RPL9D* lines were also included in this analysis. All genotypes produced approximately 11 rosette and cauline leaves and transitioned to flowering on average 25.25–27.5 days after sowing, except for *rpl9c/+ rpl9d* and *rpl9c rpl9d/+*. Both of these genotypes produced more leaves (average 12.75 and 14.25 leaves, respectively, *P* < 0.05) and *rpl9c rpl9d/+* flowered later (average 32.1 days after sowing, *P* < 0.05) than wild type (**Figures 2A,B**). Despite these differences, the rate of vegetative leaf initiation was similar for all genotypes (**Figure 2C**). During growth of the inflorescence *rpl9d* plants were not affected but *rpl9c* (*P* = 0.04), *rpl9c/+ rpl9d* (*P* < 0.005) and *rpl9c rpl9d/+* (*P* < 0.005) plants appeared to be shorter than wild type at any given time point. The growth of the two independent *rpl9c RPL9D::RPL9D* plants were not significantly different from either wild type or *rpl9c* plants indicating that RPL9D can partially complement the *rpl9c* mutation (**Figure 2D**).

The *rpl9c rpl9d* mutant was not identified in progeny of *rpl9c rpl9d/+* or *rpl9c/+ rpl9d*. Plants of these two genotypes showed 24.3% (*n* = 236) and 22.6% (*n* = 260) white seed, respectively, consistent with the double homozygous mutant being embryo lethal. By comparison, siliques of the single *rpl9c* and *rpl9d* mutants had 0% (*n* = 205) and 0.6% (*n* = 309) white seed, respectively. Examination of siliques from *rpl9c/+ rpl9d* plants showed embryos that were arrested at a late globular stage of development (**Figure 2E**). Thus RPL9 levels in the *rpl9c rpl9d* double homozygous mutant are sufficient to support early stages of embryogenesis but are not sufficient to maintain growth throughout embryogenesis.

### *RPL9* Genes in the Brassicaceae

**FIGURE 3:**
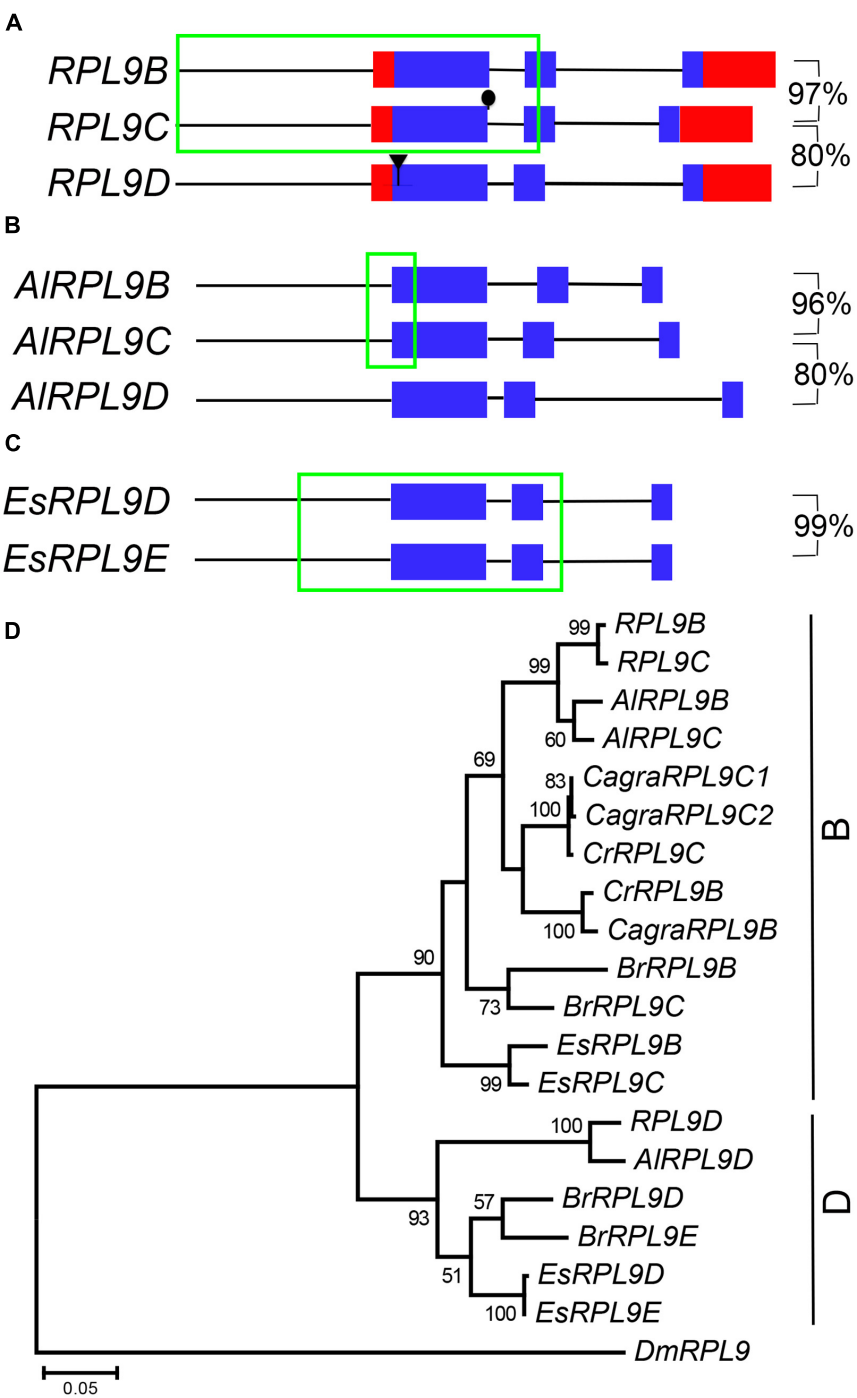
***RPL9* genes in Brassicaceae.** Diagrammatic representation of *A. thaliana* **(A)**, *Arabidopsis lyrata* **(B)**, and *Eutrema salsugineum* **(C)** genes showing UTR’s (red), exons (blue), non-coding upstream, and intron sequences (black line), and regions of 100% nucleotide sequence identity (green). Percentage nucleotide sequence identity of exons (excluding region in green box) is shown for gene pairs. Phylogenetic tree shown in **(D)** includes *A. thaliana*, *A. lyrata* (*AlRPL9*), *Capsella rubella* (*CrRPL9*), *C. grandiflora* (*CagraRPL9*), *E. salsuginum* (*EsRPL9*), *B. rapa* (*BrRPL9*), and *Drosophila melanogaster* (*DmRPL9*) genes.

*RPL9B* and *RPL9C* are tandem genes, separated by 11,136 bp on Chromosome 1. Comparison of *RPL9B* and *RPL9C* nucleotide sequence revealed a region of identical sequence extending from -927 bp upstream to +623 bp downstream of the AUG initiation codon (**Figure 3A**). This sequence included the first exon, first intron and the 5′ half of the second exon. Nucleotide sequences of the 3′ half of the second exon and the third exon diverged and were 97% identical, whereas there was no significant sequence identity between the third intron of *RPL9B* and *RPL9C*. Comparison of these two genes with *RPL9D*, on Chromosome 4, showed that *RPL9D* is more divergent with the CDS sequence sharing 80% nucleotide sequence identity with *RPL9B* and *RPL9C* (**Figure 3A**). *RPL9B/RPL9C* and *RPL9D* are located in syntenic regions of *A. thaliana* Chromosomes 1 and 4 (**Supplementary Figure S1A**). These two regions are part of a recent whole genome duplication, the α duplication, which occurred 24–40 million years ago (Simillion et al., 2002; Blanc et al., 2003; Bowers et al., 2003).

To determine whether duplicate *RPL9* genes in *A. thaliana* are conserved in closely related species we compared *RPL9* family genes from *A. lyrata*, *C. rubella, C. grandiflora, B. rapa*, and *E. salsugineum* (Hu et al., 2011; Wang et al., 2011; Slotte et al., 2013; Yang et al., 2013). *A. lyrata*, which is most closely related to *A. thaliana*, has three *RPL9* genes. We notionally designated these genes *AlRPL9B*, *AlRPL9C*, and *AlRPL9D* according to the most closely related *A. thaliana* gene (Supplementary Table S1). As in *A. thaliana, AlRPL9B*, and *AlRPL9C* are linked by 8,119 bp and share a region of identical nucleotide sequence, extending from –167 to +245 bp, which includes the 5′ region of the first exon (**Figure 3B**). The remaining coding regions of these two genes are 96% identical with no significant identity between the introns. *AlRPL9D* is more divergent and shares 80% sequence identity with *AlRPL9B.* Phylogenetic analysis showed the three genes in *A. thaliana* and *A. lyrata* fall into two distinct clades, which we named B and D group genes (**Figure 3D**).

*Capsella rubella* and *C. grandiflora* were found to have two and three *RPL9* genes, respectively. In contrast to *A. thaliana* and *A. lyrata*, the two *Capsella* species had B group but not D group genes (**Figure 3D**). The additional gene in *C. grandiflora* appeared to be due to a recent gene duplication. *CagraRPL9C1* and *CagraRPL9C2* were adjacent direct repeats differing in a single nucleotide that altered the AUG initiation codon of *CagraRPL9C2* to TTG (**Supplementary Figure S2**). This change to a non-canonical initiation codon suggests *CagraRPL9C2* may not encode a functional protein (Tikole and Sankararamakrishnan, 2006). *E. salsugineum* and *B. rapa* each had two B and two D group *RPL9* genes (**Figure 3D**). *E. salsugineum* genes occurred as pairs of tandem genes. The B group genes *EsRPL9B* and *EsRPL9C* were linked by 12,130 bp, and the D group genes *EsRPL9D* and *EsRPL9E* were linked by 9,155 bp (Supplementary Table S1). Unlike the *A. thaliana* and *A. lyrata* B group genes, *EsRPL9B* and *EsRPL9C* did not share an extended region of identical sequence. However, *EsRPL9D* and *EsRPL9E* shared identical sequence extending from -287 bp upstream to +713 bp downstream of the AUG initiation codon. The 3′ exon and intron sequences had only four base pair differences, and the third exon in these two genes shared 99% identity (**Figure 3C**).

The relationship between *A. thaliana*, *A. lyrata, C. rubella*, and *E. salsugineum RPL9* family genes was further investigated by determining whether these genes map to syntenic regions between each genome. Consistent with the phylogeny, B group genes of *A. lyrata, C. rubella* and *E. salsugineum* were in regions of synteny with *A. thaliana* Chromosome 1 carrying *RPL9B* and *RPL9C* genes (**Supplementary Figure S1A**). Synteny was also identified between chromosomal regions carrying D group genes of *A. thaliana, A. lyrata* and *E. salsugineum* (**Supplementary Figure S1B**). Notably the tandem gene pair *EsRPL9D*/*EsRPL9E* in *E. salsugineum* was located in a region of synteny with the single gene *RPL9D* in *A. thaliana* (**Supplementary Figure S1B**). This suggests that there has been either a single gene loss or gain following the divergence of *Arabidopsis* and *E. salsugineum*. Potentially the pre-α duplication genome had two tandem *RPL9* genes. Subsequent to the α duplication the *Arabidopsis* lineage has lost one *RPL9* gene.

### *RPL9* Genes in Eudicots and Monocots

**FIGURE 4:**
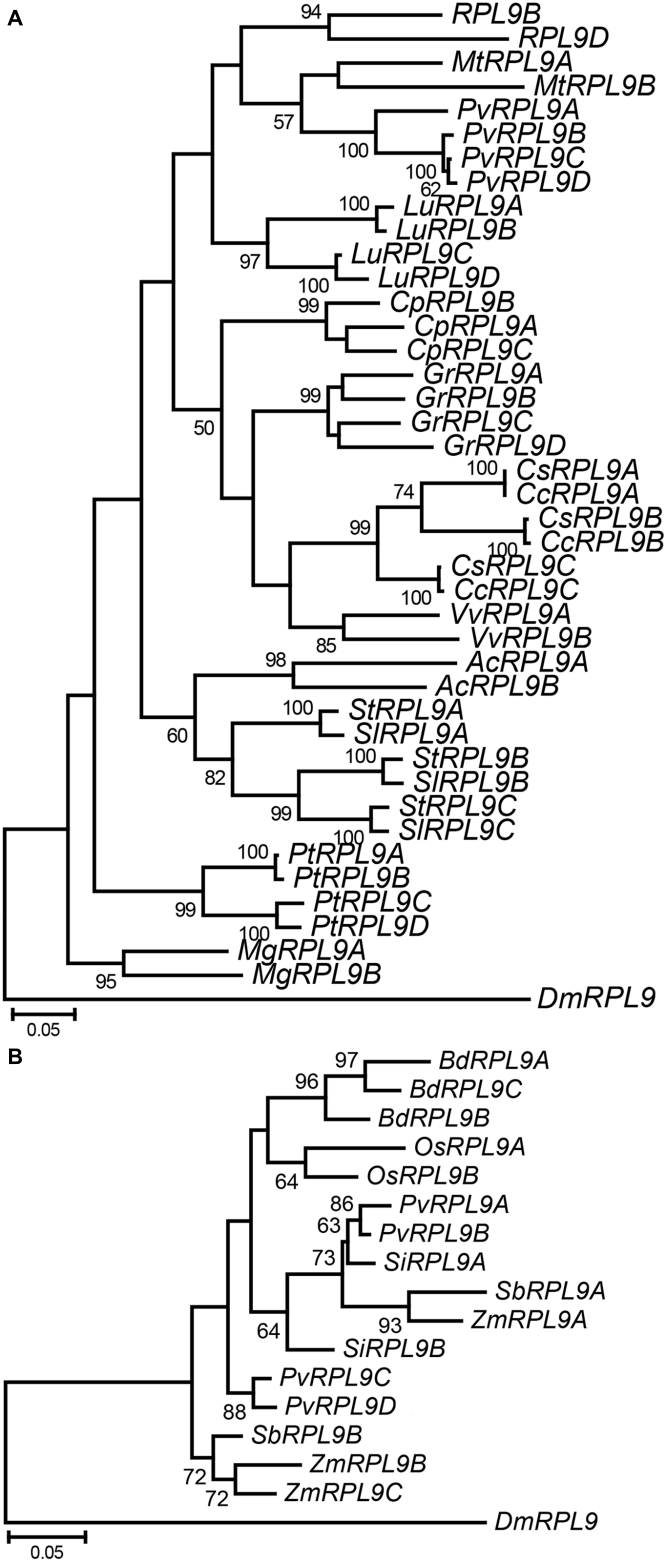
**Phylogeny of *RPL9* genes from angiosperm species.** Tree for dicot species **(A)** *Aquilegia coerulea* (*AcRPL9*)*, Carica papaya* (*CpRPL9*)*, Citrus clementina* (*CcRPL9*)*, C. sinensis* (*CsRPL9*)*, Gossypium raimondii* (*GrRPL9*)*, Linum usitatissimum* (*LuRPL9*)*, Medicago truncatula* (*MtRPL9*)*, Mimulus guttatus* (*MgRPL9*), *P. vulgaris* (*PhvRPL9*)*, Poplar trichocarpa* (*PtRPL9*)*, Solanum lycopersicum* (*SlRPL9*), *S. tuberosum* (*StRPL9*), and *Vitis vinifera* (*VvRPL9*). Tree for monocot species **(B)** *Brachypodium distachyon* (*BdRPL9*)*, Oryza sativa* (*OsRPL9*)*, Panicum virgatum* (*PvRPL9*)*, Setaria italica* (*SiRPL9*), *S. bicolor* (*SbRPL9*), and *Zea mays* (*ZmRPL9*). *D. melanogaster RPL9* gene (*DmRPL9*) is the outgroup.

To further investigate the evolution of *RPL9* families within plants we analyzed *RPL9* genes from thirteen dicot and six monocot species. These represent a diverse range of plant taxa for which whole genome sequence was available. The number of *RPL9* genes in the dicot and monocot species ranges from 2 to 4 copies (**Figure 4**). As in the Brassicaceae, tandem genes were found in the dicot species *P. tichocarpa*, *P. vulgaris*, and *Carica papaya* but were not found in the monocot species (Supplementary Table S1). Phylogenetic analysis showed that *RPL9* genes within a species tended to be more closely related to each other than to orthologs in distantly related species. The exceptions were for closely related species. The two *Citrus* and two *Solanum* species had one gene in each of three clusters (**Figure 4A**). The closely related monocots *Z. mays* and *S. bicolor*, had *RPL9* genes in two separate clusters (**Figure 4B**). The estimated gene trees suggest that *RPL9* family members undergo homogenization over time, leading to limited variation between genes within a species and greater variation of gene families between species. This lack of concordance between species and gene trees is a hallmark of concerted evolution (Arguello and Connallon, 2011).

### *RPL4, RPL5*, *RPL27a, RPL36a*, and *RPS6* Genes in the Brassicaceae

We have shown that *RPL9* family genes have largely been retained during the divergence of the Brassicaceae but there is also evidence of rearrangements since divergence of different species. To investigate whether such rearrangements are common to other ribosomal protein gene families we examined phylogenetic relationships between members of five other ribosomal protein gene families in the Brassicaceae. The gene families selected for analysis included genes encoding RPL4, RPL5, RPL27a, RPL36a, and RPS6. In *A. thaliana*, each of these ribosomal proteins is encoded by two functional and redundant genes (Yao et al., 2008; Fujikura et al., 2009; Creff et al., 2010; Rosado et al., 2010; Casanova-Sáez et al., 2014; Zsögön et al., 2014).

**FIGURE 5:**
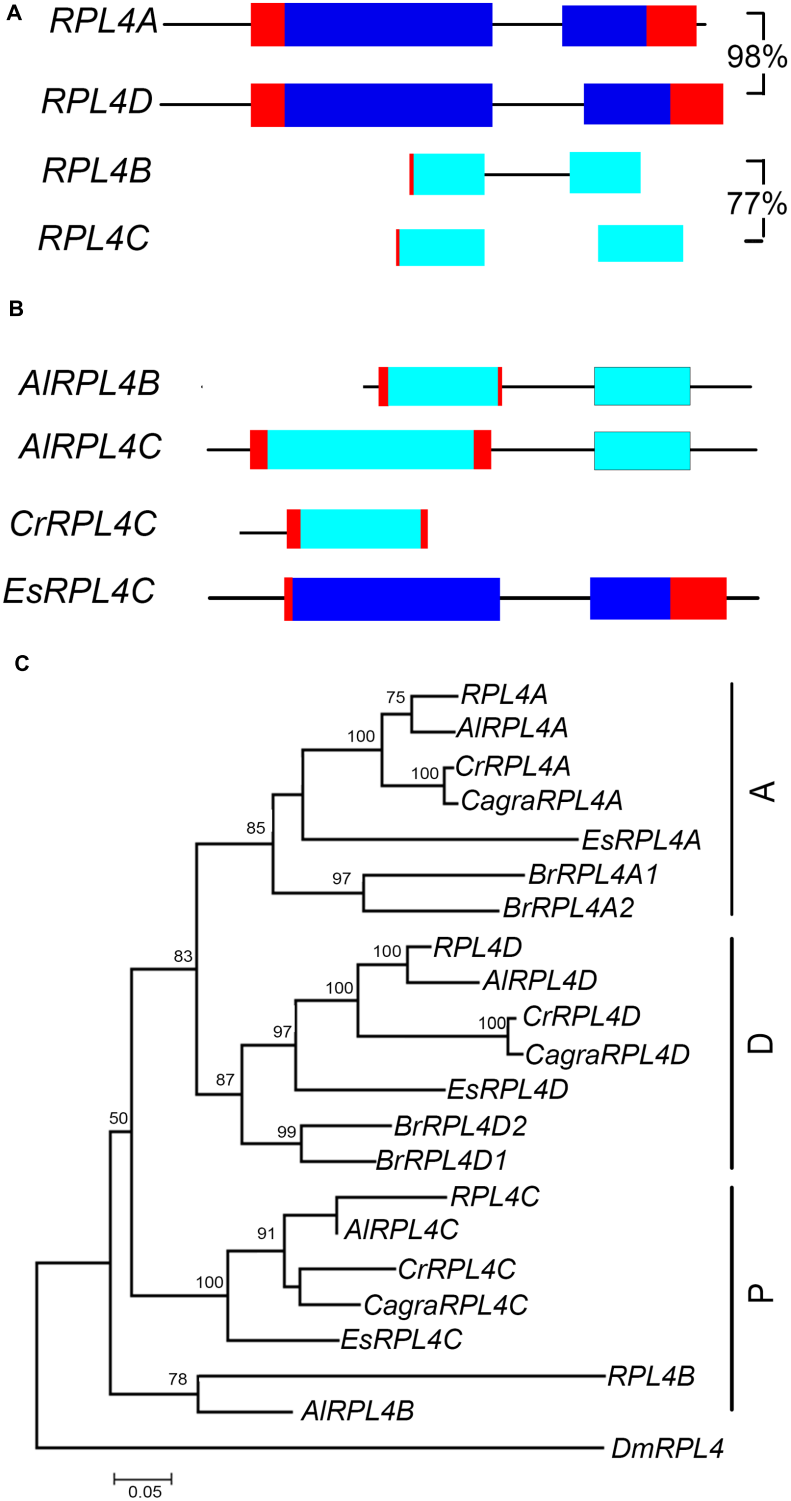
***RPL4* genes in Brassicaceae.** Diagrammatic representation of *A. thaliana* A group **(A)** and P group genes **(B)** showing UTR’s (red), exons (blue), non-coding upstream, and intron sequences (black line), and putative non-functional coding sequences (light blue). Percentage nucleotide sequence identity of exons is shown for *A. thaliana* genes and pseudogene pair. Phylogenetic tree of *RPL4* genes in Brassicaceae **(C)** includes genes of species listed in **Figure 3**. The *C. grandiflora RPL4C* gene sequence used in phylogenetic analysis is likely incomplete.

The *RPL4* family in *A. thaliana* comprises two functional genes, *RPL4A* and *RPL4D*, which are in syntenic regions retained from the α duplication (Bowers et al., 2003; Rosado et al., 2010) (**Figure 5A**, **Supplementary Figure S3a**). Phylogenetic analysis showed that *A. lyrata, C. rubella, C. grandiflora*, and *E. salsuginum* had one gene that clustered with *RPL4A* and another gene that clustered with *RPL4D*, forming A and D groups (**Figure 5C**). *B. rapa* had two genes in each of these groups. Analysis of *A. thaliana, A. lyrata*, and *C. rubella* chromosomal regions carrying A or D group genes showed these genes were in regions sharing synteny, indicating retention of A and D group genes in these species following the α duplication (**Supplementary Figure S3a**). The *RPL4* family in *A. thaliana* also includes two pseudogenes, *RPL4B* and *RPL4C*, which comprise partial sequences (Barakat et al., 2001) (**Figure 5A**). *RPL4* pseudogenes were also found in *A. lyrata* and *C. rubella* species (**Figure 5B**). The pseudogenes formed distinct clusters, which we designated P group genes (**Figure 5C**). In the P group, a single gene, *EsRPL4C*, was full length and was predicted to encode a functional protein (**Supplementary Figure S4**). Although some P group genes occurred in regions of synteny, *RPL4B, RPL4C, AlRPL4B*, and *CrRPL4C* retained different *RPL4* sequences, and *AlRPL4C* had a base change that generated a premature stop codon (**Supplementary Figures S3b** and **S4**). This suggests the pseudogenes were derived from independent events.

**FIGURE 6:**
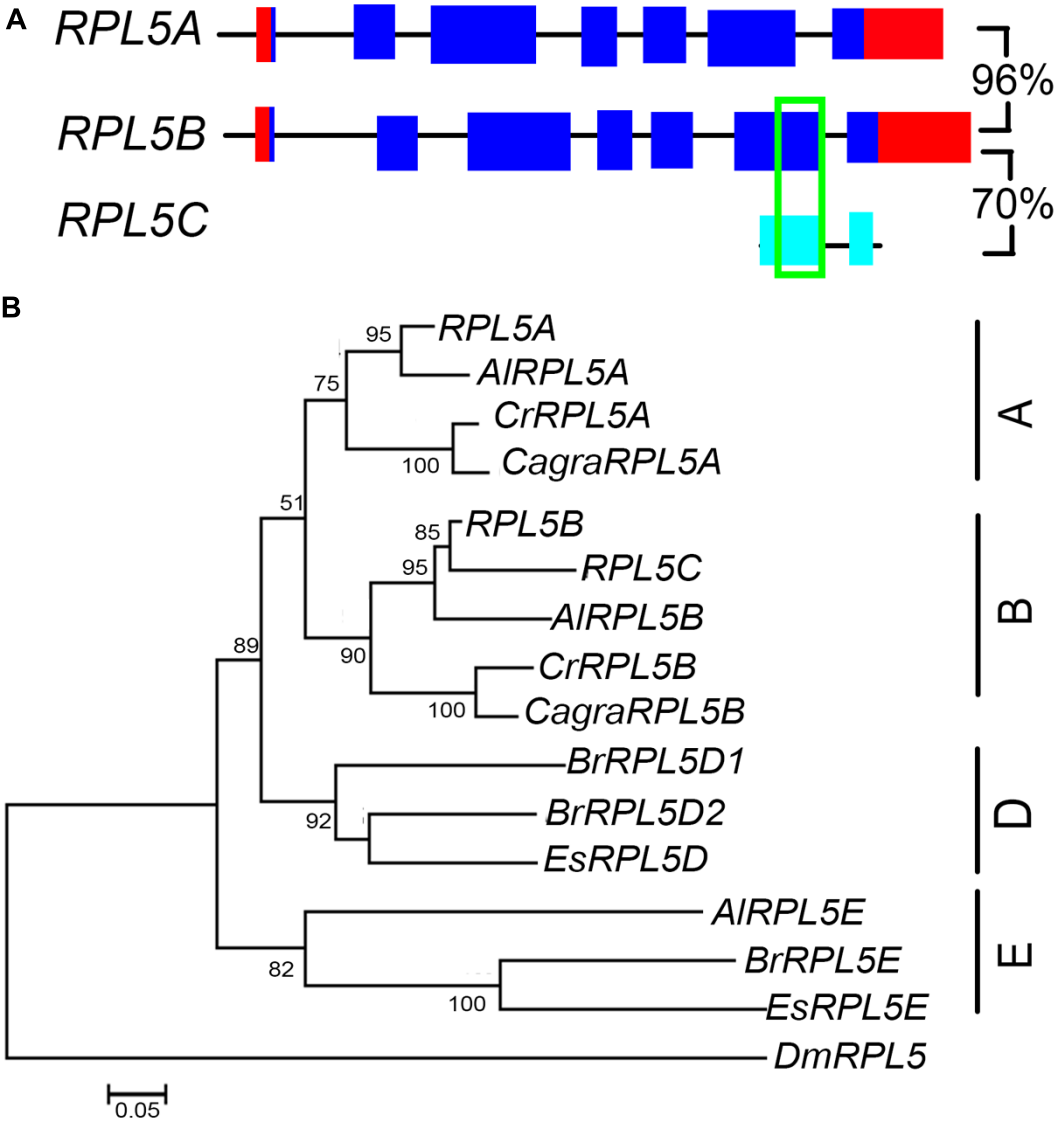
***RPL5* genes in Brassicaceae.** Diagrammatic representation of *A. thaliana RPL5* genes **(A)** showing UTR’s (red), exons (blue), non-coding upstream, and intron sequences (black line), exons of pseudogenes (light blue) and regions of 100% nucleotide identity (green). Percentage nucleotide sequence identity of exons is shown for genes and pseudogene. The phylogenetic tree of *RPL5* genes in Brassicaceae **(B)** includes genes of species as listed in **Figure 3**.

*A. thaliana* has two functional *RPL5* genes, *RPL5A* (also known as *ATL5*, *PIGGYBACK3*, *ASYMMETRIC LEAVES1/2 ENHANCER6*, *OLIGOCELLULA5)* and *RPL5B* (also known as *OLIGOCELLULA7*), as well as one pseudogene, *RPL5C* (Barakat et al., 2001; Pinon et al., 2008; Yao et al., 2008; Fujikura et al., 2009). Comparison of *RPL5B* and the pseudogene showed that they shared a 109 bp region of identical nucleotide sequence (**Figure 6A**). Unlike *RPL9* and *RPL4*, the *RPL5* genes were not in syntenic regions of the *A. thaliana* genome, indicating these genes have not been retained from a recent genome duplication or that synteny in these chromosomal regions has been lost since genome duplication. Phylogenetic analysis showed that *A. lyrata*, *C. rubella*, and *C. grandiflora* had one gene that clustered with *RPL5A* and one gene that clustered with *RPL5B*, forming A and B groups (**Figure 6B**). The *A. thaliana, A. lyrata*, and *C. rubella* genes in each group were in regions of synteny indicating a common origin (**Supplementary Figures S5A,B**). A third *A. lyrata* gene, *AlRPL5E*, two *E. salsuginum*, and three *B. rapa* genes clustered into a group that was distinct from genes in *Arabidopsis* and *Capsella* species (**Figure 6B**). *AlRPL5E* had no apparent ortholog in *A. thaliana* although *AlRPL5E* was located in a region of synteny with *RPL5A* (**Supplementary Figure S5A**).

The phylogeny of *RPL27a* showed two gene clusters. All species had a single gene in each cluster, except *B. rapa* where *RPL27a* genes were more closely related to each other than to *RPL27a* genes in the other Brassicaceae species (**Supplementary Figure S6A**). In *A. thaliana*, the *RPL36a* family comprises *RPL36aA* and *RPL36aB* (also known as *APICULATA2*) (Barakat et al., 2001; Casanova-Sáez et al., 2014). Each Brassicaceae species had a *RPL36a* family member that clustered with *RPL36aA* and a member that clustered with *RPL36aB*, except for *C. grandiflora*, which only had one *RPL36a* gene, and *B. rapa*, which had several genes in each cluster (**Supplementary Figure S6B**). Likewise, *RPS6* genes clustered into A and B groups and all species had a single gene in each group, with two exceptions. *A. lyrata* had no A group and two B group genes, and *B. rapa* had multiple genes in each group (**Supplementary Figure S6C**).

In summary, the phylogenies of *RPL4, RPL5*, *RPL27a, RPL36a*, and *RPS6* ribosomal protein gene families show evidence of retention of genes following whole genome duplication. However, there is also evidence of gene gain and loss, partial gene loss, and some instances concerted evolution of genes within a species.

## Discussion

All ribosomal proteins in *A. thaliana* are encoded by small gene families. Typically members of a family encode proteins sharing 95–100% identity, although there are several exceptions where family members encode proteins that show as little as 70% amino acid identity (Barakat et al., 2001). Family members may be required to maintain the dose of a ribosomal protein or each member may encode a variant of a ribosomal protein that contributes to production of functionally heterogeneous ribosome populations (Chang et al., 2005; Carroll et al., 2007; Byrne, 2009; Horiguchi et al., 2012; Xue and Barna, 2012). *RPL9C* encodes a protein that shares 89% amino acid identity to the *RPL9D* encoded protein. Despite this divergence, genetic analysis indicates *RPL9C* and *RPL9D* are redundant. Firstly, mutation in *RPL9D* enhanced *rpl9c*, and conversely, mutation in *RPL9C* enhanced the phenotype of *rpl9d*. Secondly, increasing expression of *RPL9D*, by an *RPL9D:RPL9D* transgene, repressed the *rpl9c* mutant. Thirdly, the *rpl9c rpl9d* double homozygous mutant arrested at the globular stage of development. Embryo arrest at the globular stage is similar to the phenotype of *aml1*, and to *embryo-defective* (*emb*) mutants *emb2167* and *emb2296*, which correspond to mutations in cytoplasmic ribosomal protein genes *RPL8A* and *RPL40B* (Weijers et al., 2001; Tzafrir et al., 2004; Meinke, 2013). Deficiency of any one ribosomal protein impairs ribosome assembly or results in inefficient formation of translation-competent ribosomes (de la Cruz et al., 2015). As such it is predicted that inadequate levels of RPL9 lead to a reduction in ribosome production and impairment of translation.

Whole genome duplication is common in flowering plants and several duplications have occurred in dicot and monocot lineages (Bowers et al., 2003; Paterson et al., 2004). Genes encoding proteins that are dosage sensitive, such as transcription factors, signaling pathway, proteasome and ribosomal protein genes are preferentially retained following whole genome duplication (Freeling, 2009). The most recent genome duplication in the rosids, the α duplication, occurred prior to the *Arabidopsis-Brassica* split (Simillion et al., 2002; Blanc et al., 2003; Bowers et al., 2003). The phylogenetic trees of *RPL9, RPL4, RPL5*, *RPL27a, RPL36a*, and *RPS6* families in the Brassicaceae showed that genes clustered into two main groups, consistent with retention of ribosomal protein genes following the α duplication. Furthermore, *RPL9*, *RPL4*, and *RPL5* genes within a group map to regions of synteny indicating retention following genome duplication. However, there were exceptions in which closely related species varied in ribosomal protein gene copy number indicating recent gain or loss of family members. For example, an additional *RPL9* gene in *E. salsugineum* suggested loss of one *RPL9* gene after the divergence of the Camelineae species and *E. salsugineum.* Further *RPL9* gene loss and gene duplication appears to have occurred in *Capsella* species. Compared with *A. thaliana*, *A. lyrata* had an additional *RPL5* gene. *B. rapa* has undergone a recent whole genome triplication following divergence from *A. thaliana* (Wang et al., 2011). Retention of ribosomal protein genes following genome triplication would predict *B. rapa* to have six members of each ribosomal protein compared to two members in other Brassicaceae. All ribosomal protein families examined showed *B. rapa* genes occurred in higher copy number indicating that these genes have been retained following genome triplication. However, all families had fewer than six genes indicating a tendency toward loss of ribosomal protein genes. Pseudogenes resulting from partial gene deletion were present in several families and were most notable in the *RPL4* family. Surprisingly *RPL4* pseudogenes appeared to have been generated through independent deletion events. Potentially these genes are in chromosomal regions subject to frequent rearrangements.

Phylogenies of *RPL9* genes in distantly related dicot and monocot species showed clustering of genes within a species rather than between species. A trend where genes within a species are closely related and cluster in a phylogenetic tree was also evident for some *RPL5* and *RPL27a* genes in the Brassicaceae. Such gene relationships indicate ribosomal protein genes in plants undergo concerted evolution (Arguello and Connallon, 2011). Furthermore *RPL9* genes in *A. thaliana*, *A. lyrata*, and *E. salsuginum* showed extended regions of identical nucleotide sequence characteristic of recent gene conversion events through homologous recombination between tandem copies of ribosomal protein genes (Chen et al., 2007). Concerted evolution of ribosomal protein genes is also observed in *Saccharomyces cereviseae* and closely related yeast species (Gao and Innan, 2004). Gene conversion could serve to maintain conservation of proteins that contribute to a complex macromolecule. In this case, amino acids that have low functional significance in a ribosomal protein would vary between between plant species. Interestingly, tandemly arrayed ribosomal RNA genes also undergo concerted evolution and maintain a high level of sequence homogeneity in eukaryotes (Brown et al., 1972; Copenhaver and Pikaard, 1996; Gonzalez and Sylvester, 2001; Stage and Eickbush, 2007). Potentially, ribosomal protein genes and rRNA co-evolve in order to maintain optimal RNA-protein interactions in the ribosome and limit synthesis of inefficient ribosomes (Roberts et al., 2008).

Ribosomal protein gene copy number in plants appears to be under constraint consistent with the gene balance hypothesis. Mechanisms maintaining gene copy number involves retention of paralogs following whole genome duplication. However, partial or whole gene deletion, tandem duplication and gene conversion are prominent features of ribosomal protein gene families across species, reflecting dynamic evolution of these genes.

## Author Contributions

DD, SF, ZL, and MB carried out the experiments, prepared the figures, and reviewed the manuscript. MB wrote the manuscript.

## Conflict of Interest Statement

The authors declare that the research was conducted in the absence of any commercial or financial relationships that could be construed as a potential conflict of interest.

## Supplementary Material

**FIGURE S1 |*RPL9* genes in Brassicaceae occur in regions of synteny.** CoGe outputs showing chromosomal regions of synteny. Region of *A. thaliana* Chromosome 1, around *RPL9B* and *RPL9C*, aligned with the region of Chromosome 4 carrying *RPL9D*, *A. lyrata* region around *AlRPL9B* and *AlRPL9C*, and *E. salsuginum* region around *EsRPL9B* and *EsRPL9C* **(A)**. Region of *A. thaliana RPL9D* aligned with *A. lyrata* region around *AlRPL9D* and *E. salsuginum* region around *EsRPL9D* and *EsRPL9E* **(B)**. Chromosomes are indicated as dotted black lines with genes (green) indicated on both Watson and Crick strands. Unsequenced regions are marked (orange). Regions of identified sequence similarity (pink) are shown above the chromosomes. Interconnecting lines between chromosomes (pink) mark *RPL9* genes. **FIGURE S2 | Tandem *C. grandiflora RPL9* genes.** Nucleotide sequence of *CagraRPL9C1* (solid underline) and *CagraRPL9C2* (dotted underline) genes. Exons (orange) and introns (black, lower case) are shown with initiation codons highlighted in green and stop codons highlighted in yellow. **FIGURE S3 |*RPL4* genes in Brassicaceae occur in regions of synteny.** CoGe outputs showing regions of synteny around *A. thaliana RPL4D* and *RPL4A*, *A. thaliana RPL4D* and *A. lyrata AlRPL4D* and *A. thaliana RPL4D*, and *C. rubella CrRPL4D* **(A)**, and regions of synteny around *A. thaliana RPL4C* and *A. lyrata AlRPL4C*, and *A. thaliana RPL4C* and *C. rubella CrRPL4C* **(B)**. Chromosomes are indicated as dotted black lines with genes (green) indicated on both Watson and Crick strands. Regions of identified sequence similarity (pink) are shown above the chromosomes. Interconnecting lines between chromosomes (pink) mark *RPL4* genes. **FIGURE S4 | *RPL4* pseudogenes in Brassicaceae.** Alignment of nucleotide sequences of P group genes. Start and stop codons are highlighted in red. The *C. grandiflora RPL4C* gene sequence is likely incomplete. **FIGURE S5 |*RPL5* genes in Brassicaceae occur in regions of synteny.** CoGe outputs showing regions of synteny around *A. thaliana RPL5A* **(A)** and *RPL5B* **(B)** genes with *RPL5* genes in *A. lyrata* and *C. rubella.* Chromosomes are indicated as dotted black lines with genes (green) indicated on both Watson and Crick strands. Regions of identified sequence similarity (pink) are shown above the chromosomes. Interconnecting lines between chromosomes (pink) mark *RPL5* genes. **FIGURE S6 | Phylogenetic trees of *RPL27a, RPL36a*, and *RPS6* genes in Brassicaceae.** Phylogenetic trees of *RPL27a* **(A)***, RPL36a* **(B),** and *RPS6* **(C)** includes *A. thaliana*, *A. lyrata*, *C. rubella*, *C. grandiflora*, *E. salsuginum*, *B. rapa*, and *D. melanogaster* genes.
