# Supplementary material for: Dosage Sensitivity of RPL9 and Concerted Evolution of Ribosomal Protein Genes in Plants
Source: Front Plant Sci. 2015 Dec 16;6:1102. doi: 10.3389/fpls.2015.01102 (PMC4679983; doi:10.3389/fpls.2015.01102)
Supplement: Supplementary file 1 [file Image_1.PDF]

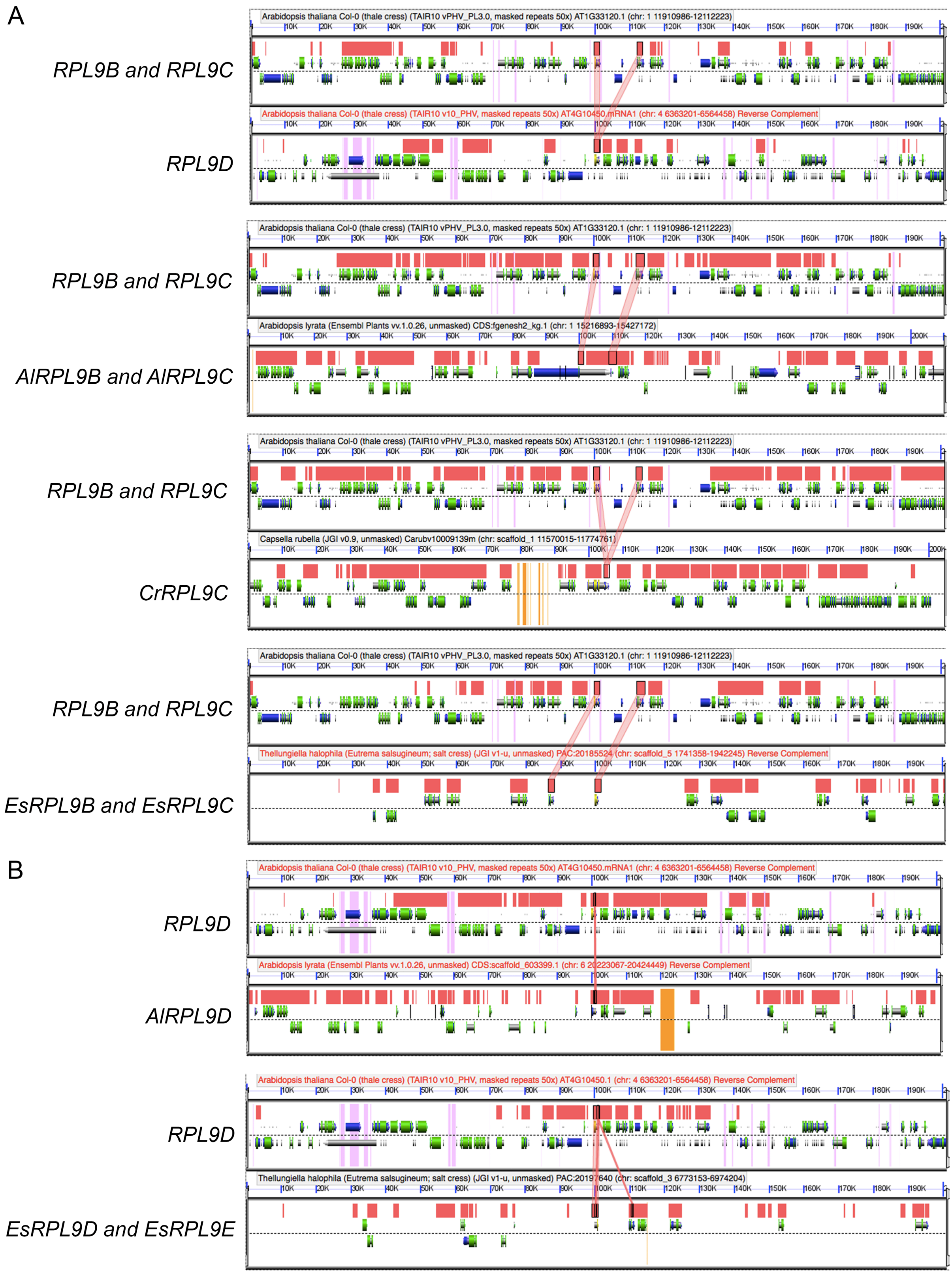
A
RPL9B and RPL9C
Arabidopsis thaliana Col-0 (thale cress) (TAIR10 vPHV_PL3.0, masked repeats 50x) AT1G33120.1 (chr: 1 11910986-12112223)
RPL9D
Arabidopsis thaliana Col-0 (thale cress) (TAIR10 v10_PHV, masked repeats 50x) AT4G10450.mRNA1 (chr: 4 6363201-6564458) Reverse Complement
RPL9B and RPL9C
AlRPL9B and AlRPL9C
Arabidopsis lyrata (Ensembl Plants vv.1.0.26, unmasked) CDS:fgenesh2_kg.1 (chr: 1 15216893-15427172)
RPL9B and RPL9C
CrRPL9C
Capsella rubella (JGI v0.9, unmasked) Carubv10009139m (chr: scaffold_1 11570015-11774761)
RPL9B and RPL9C
EsRPL9B and EsRPL9C
Thellungiella halophila (Eutrema salsugineum; salt cress) (JGI v1-u, unmasked) PAC:20185524 (chr: scaffold_5 1741358-1942245) Reverse Complement
B
RPL9D
AlRPL9D
Arabidopsis lyrata (Ensembl Plants vv.1.0.26, unmasked) CDS:scaffold_603399.1 (chr: 6 20223067-20424449) Reverse Complement
RPL9D
Arabidopsis thaliana Col-0 (thale cress) (TAIR10 v10_PHV, masked repeats 50x) AT4G10450.1 (chr: 4 6363201-6564458) Reverse Complement
EsRPL9D and EsRPL9E
Thellungiella halophila (Eutrema salsugineum; salt cress) (JGI v1-u, unmasked) PAC:20197640 (chr: scaffold_3 6773153-6974204)

Figure S1

```
>Cagra.18493s0001 | Scaffold18493:5902..6971 forward

NNNNNNNNNNNNNNNNNNNNNNNNNNNNNNNNNNNNNNNNNNNNNNNNTTCTTCCACCCT
TTAAAAAGCCGCCGTCAATACATTTTGTTAGGGTTTCAGCCTTTGATCAAACCAGAGCAG
CGCGAGAGTCGTCGTCGTGAGAGAGAGCAAAACCAAAGGCCGGAGAAGATGAAGACTATT
CTATCTTCCGAGACGATGGACATCCCCGACACCGTAACCATCAAGGTTCACGCCAAAGTG
ATCGAAGTGGAAGGTCCTCGCGGCAAGCTTGTTCGCGATTTCAAGCATCTCAACCTCGAT
TTCCAGCTGATCAAGGACCCCGAGACTGGGAAGAAGAAGCTTAAGATCGACTCGTGGTTC
GGGTCTCGCAAAACCAGTGCTTCCATCAGAACTGCTCTTAGCCACGTTGATAACCTCATC
TCCGGTGTTACCAGAGGTTTCAGATACAAGATGAGGTTTGTGTATGCTCATTTTCCCATC
AACGCTTCCATTGGTGGTGATGGCAAGTCCATTGAGATCCGTAACTTCCTTGGCGAGAAG
AAGgtttgtatttcaatagatctcggaatgatatagagtgacactagattcgattcttgg
aaattgtgtatactaatcgatgattgattgatttcatggtttgtgaatactgaacttgtt
ttgaaatgtggttattggaatgatagGTGAGGAAGGTAGATATGTTGGATGGTGTAACCA
TTGTTCGATCTGAGAAGGTGAAGGATGAGATTGTTCTTGATGGTAATGACATTGAGCTTG
TTTCAAGGTCATGCGCTTTGATCAATCAGgtactctctttcagatacaatatagagactt
gtgttaagtagtctttgttgatgtttgaggtctgtaaatggattgattcatctgaaactg
aaatgtaaaatgatttgtacgatcgacattgttatctcgaagaatgttcaataagctctg
gccaagttttgcatattgtttcatatgtcttaggctttcaatggtaaggtttcaatctca
caatggttaattgtaatgtgtagAAATGTCACGTAAAGAAGAAGGATATCAGGAAGTTTC
TTGACGGTATCTATGTCAGCGAGAAGAGCAAGATCGTCGAAGAGGAATAGTTTTATTTGA
TCGTCATCATAGCTCAATTTTCTCTATTTTGAAGACTATTCTATCTTCCGAGACGATGGA
CATCCCCGACACCGTAACCATCAAGGTTCACGCCAAAGTGATCGAAGTGGAAGGTCCTCG
CGGCAAGCTTGTTCGCGATTTCAAGCATCTCAACCTCGATTTCCAGCTGATCAAGGACCC
CGAGACTGGGAAGAAGAAGCTTAAGATCGACTCGTGGTTCGGGTCTCGCAAAACCAGTGC
TTCCATCAGAACTGCTCTTAGCCACGTTGATAACCTCATCTCCGGTGTTACCAGAGGTTT
CAGATACAAGATGAGGTTTGTGTATGCTCATTTTCCCATCAACGCTTCCATTGGTGGTGA
TGGCAAGTCCATTGAGATCCGTAACTTCCTTGGCGAGAAGAAGgtttgtatttcaataga
tctcggaatgatatagagtgacactagattcgattcttggaaattgtgtatactaatcga
tgattgattgatttcatggtttgtgaatactgaacttgttttgaaatgtggttattggaa
tgatagGTGAGGAAGGTAGATATGTTGGATGGTGTAACCATTGTTCGATCTGAGAAGGTG
AAGGATGAGATTGTTCTTGATGGTAATGACATTGAGCTTGTTTCAAGGTCATGCGCTTTG
ATCAATCAGgtactctctttcagatacaatatagagacttgtgttaagtagtctttgttg
atgtttgaggtctgtaaatggattgattcatctgaaactgaaatgtaaaatgatttgtac
gatcgacattgttatctcgaagaatgttcaataagctctggccaagttttgcatattgtt
tcatatgtcttaggctttcaatggtaaggtttcaatctcacaatggttaattgtaatgtg
tagAAATGTCACGTAAAGAAGAAGGATATCAGGAAGTTTCTTGACGGTATCTATGTCAGC
GAGAAGAGCAAGATCGTCGAAGAGGAATAGTTTTATTTGATCGTCATCATAGCTCAATTT
TCTCTATTTNNNNNNNNNNNNNNNNNNNNNNNNNNNNNNN
```

Figure S2

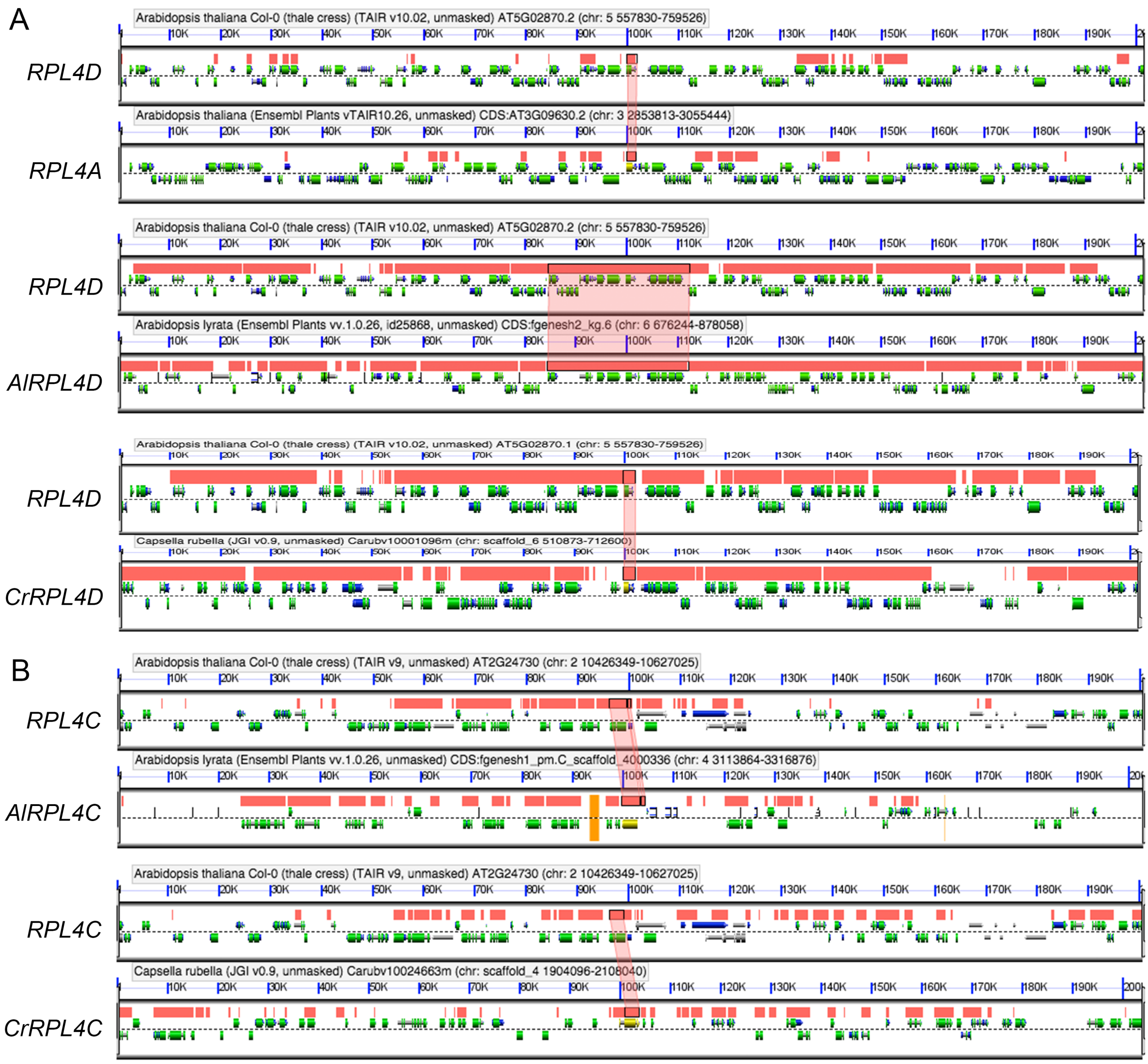

A
Arabidopsis thaliana Col-0 (thale cress) (TAIR v10.02, unmasked) AT5G02870.2 (chr: 5 557830-759526)
RPL4D
Arabidopsis thaliana (Ensembl Plants vTAIR10.26, unmasked) CDS:AT3G09630.2 (chr: 3 2853813-3055444)
RPL4A
Arabidopsis thaliana Col-0 (thale cress) (TAIR v10.02, unmasked) AT5G02870.2 (chr: 5 557830-759526)
RPL4D
Arabidopsis lyrata (Ensembl Plants vv.1.0.26, id25868, unmasked) CDS:fgenesh2_kg.6 (chr: 6 676244-878058)
AlRPL4D
Arabidopsis thaliana Col-0 (thale cress) (TAIR v10.02, unmasked) AT5G02870.1 (chr: 5 557830-759526)
RPL4D
Capsella rubella (JGI v0.9, unmasked) Carubv10001096m (chr: scaffold_6 510873-712600)
CrRPL4D
B
Arabidopsis thaliana Col-0 (thale cress) (TAIR v9, unmasked) AT2G24730 (chr: 2 10426349-10627025)
RPL4C
Arabidopsis lyrata (Ensembl Plants vv.1.0.26, unmasked) CDS:fgenesh1_pm.C_scaffold_4000336 (chr: 4 3113864-3316876)
AlRPL4C
Arabidopsis thaliana Col-0 (thale cress) (TAIR v9, unmasked) AT2G24730 (chr: 2 10426349-10627025)
RPL4C
Capsella rubella (JGI v0.9, unmasked) Carubv10024663m (chr: scaffold_4 1904096-2108040)
CrRPL4C

Figure S3

Figure S4

```
AlRPL4C     1                                                          ATGTCCACC

RPL4D       1      ATGGTTGCCTCAGCCGCCGCACGACCTCTTGTCACCGTCCAAGGACTTGACGGTGACATG
AlRPL4C     10     GCCACCGCCACCGCCGACGTTCGTCCTCTAGTAACCGTCCAATCTCTGGACGGCGATATG
EsRPL4C     1      ATGGCTGCC   GCTGCCGTTCGCCCTCTAGTTACCATCCAATCTCTGGAAGGTGATATG

RPL4D       61     AGCACCGATCAATCCACCACCGTCACTTTACCAGACGTCATGACTGCTCCAGTTCGACCT
AlRPL4C     64     ATCACAGATCAAACCACCACACTACCTCTCCCCGATGTAATGAAAGCACCAGTTCGTCCC
CrRPL4C     1                             ATGTATCACAGATCAAACCACCACACTACCTCTCCCC
EsRPL4C     58     AGCACTGATCAAGCCACAACTATACCTCTTCCCGACGTAATGAAAGCTCCGGTTCGTCCC

RPL4D       121    GACATTGTCAACTTCGTCCACGCCCAAATCTCCAACAACAGCCGTCAGCCTTACGCAGTC
AlRPL4C     124    GACGTTGTATCCCATATCCACGCTCAGATATCAAACAACAGTCGCCAACCTTACGCCGTC
CagraRPL4C  1                                             ATGCCCGGGGCTATTCGGACT
CrRPL5A     37     GATGTAATGAAAGCACCAGTTCGTCCCGACGTTGTATCCCATATCCACGCTCAGATATCA
EsRPL4C     118    GACGTCGTGAACCATGTCCACGCTCAGATCTCAAACAACAGTCGCCAACCTTACGCTGTC

RPL4D       181    TCCAAAAAGGCCGGTCACCAAACCTCCGCCGAGTCCTGGGGAACCGGAAGAGCCGTGTCA
AlRPL4C     184    TCAAAACACGCTGGTCATCAAACTTCCGCCGAGTCTTGGGGTACAGGACGAGCCGTATCA
CagraRPL4C  123    CCGGAGCCTTTTACAAGAGAAATAGCCGCCGCCGTCGCCGCCGTTCGTACTCTAGTATCA
CrRPL4C     132    AACAACAGTCGCCAACCTTACGCCGTCTCAAAACACGCTGGTCATCAAACTTCCGCCGAG
EsRPL4C     178    TCGAAGAAAGCCGGTCATCAGACTTCAGCCGAATCGTGGGGTACAGGACGTGCCGTGTCT

RPL4D       241    CGTATCCCTCGTGTTCCTGGTGGTGGAACTCACCGTGCCGGTCAAGCAGCGTTCGGAAAC
AlRPL4C     244    CGTATCCCTCGTGTTGCCGGAGGTGGTACTCACCGTGCCGGTCAAGCAGCTTTGGGAAAC
CagraRPL4C  196    CGTATCCCTCGTGTCCCTGGAGGTTGTACTCACCGTGCCGGTCAAGCGGCTTTCGGAAAC
CrRPL4C     193    TCTTGGGGTACAGGACGAGCCGGTTGTACTCACCGTGCCGGTCAAGTGGCTTTCGGAAAC
EsRPL4C     238    CGTATCCCTCGTATTCCCGGAGGCGGAACTCACCGTGCCGGTCAAGCCGCGTTCGGAAAC

RPL4D       301    ATGTGTCGTGGTGGTCGTATGTTTGCTCCGACTAAGATCTGGAGACGCTGGCACCGTCGT
AlRPL4B     1      ATGTGTCGTGGTGGTCGAATGTTTGCTCCGACTAAAATCTGGCGACGGTTACATTGCCGT
AlRPL4C     304    ATGTGTCGTAGTGGACGAATGTTTGCTCCGACGAAGACTTACCGTCGTTGGCACCGTCGT
CagraRPL4C  256    ATGTGTCGTGGTGGTCGTATGTTTGCTCCGACGAAGCCTTACCGTCACTGGCACCGTAGT
CrRPL4C     253    ATGTGTCGTGGTTGTCGTATGTTTGCTCTGACGAAGATCTACCGACGCTGGCACCGTAGT
EsRPL4C     298    ATGTGTCGCGGTGGTCGGATGTTTGCTCCGACTAAGATCTGGCGTCGTTGGCACCGTCGC

RPL4D       361    GTCAATGTCAACATGAAGAGGCACGCGATTGTTTCTGCAATCGCTGCTACTGCTGTTCCA
AlRPL4B     61     GTTAATGTCAATATAAAGAGACACGCCATGGTTTCCGCAATCGCTGCTACCGCAGTTCCG
AlRPL4C     364    GTCAATAGCAATTTGAAACGACACGCGATTGTATCAGCGATTGCTGCGACTTCGG-
CagraRPL4C  316    GTCAATGTCAATTTGAAACGGCACGCGATTATT
CrRPL4C     313    GTCAATCTCAATTTGAAACGGCACGCGATCGTATCGGCGATTGCTGCGACTTCTGTTCCG
EsRPL4C     358    GTCAATGTCAATTTGAAGAGACACGCGATCGTTTCCGCAATTGCCGCGACTGCTGTTCCT

RPL4D       421    GCTCTTGTGATGGCTCGTGGTCACAAGATCGAGAATGTTCCTGAGATGCCTCTTGTTGTT
AlRPL4B     121    TCTCTAGTAATGGCTCGTGGTCACAAGATTGAGAATGTTCCAGAGTTCCCGCTTGTAGTT
AlRPL4C     419     CTTTAGTGATGGCTCGTGGACATAAGATTGAGAATCTTCCTGAGTTTCCTTTAGTGGTT
CagraRPL4C  346                               ATTGAGAATGTTCCTGAGTTTCCTCTTGTGGTT
CrRPL4C     373    GCTCTTTTGATGGCACGTGGTCATATGATTGAGAATGTTCCTGAGCTTCCTCTTGTGGT
EsRPL4C     418    GCTCTTGTGATGGCTCGTGGTCATCGGATCGAGAATGTTCCTGAGATGCCTTTGGTGGTT

RPL4D       481    AGCGACTCAGCTGAAGCTGTGGAGAAGACATCAGCTGCGATCAAGGTATTGAAGCAGATC
AlRPL4B     181    GGTGATTCGATTGAATCCGTTGAGGAAACTTCTGAGGCTGTTAATGTTTTGAAGCGGATC
AlRPL4C     478    AGTGATTATGTTGAGTCTGTTGAGAAAACTAGTGATGCGGTTAAGGTTTTGCAACAGATT
CagraRPL4C  379    AGTGATTCAGCTGTGTCTGTTGAGAAGAC
EsRPL4C     478    AGTGACTCTATCGAGTCTGTGGAGAAGACGAGTGGTGCGATCAAGGTTTTGGAGCAGATC

RPL4D       541    GGTGCTTACGACGATGCGGAGAAAGCTAAGAACAGTATTGGAATTCGTCCTGGTAAAGGT
AlRPL4B     241    GGTGCTTTCGCTGACGTAGAGAAGGCGAAAGATAGTGTTTGAGTTCGTTCTGGTAAAGGT
AlRPL4C     538    GGTGCTTATGCTGATGCATAGAAGGCTAAAGATAGTATTGGGATTAGGTCTGGTGTAGGT
EsRPL4C     538    GGTGCTTACCCTGATGCTGAGAAGGCGAAAAATAGTATGGGGATTCGATCTGGTGTAGGT

RPL4D       601    AAAATGAGGAATCGTCGTTACATTTCTAGGAAAGGTCCTCTTGTTGTGTTTGGAACTGAA
AlRPL4B     301    AAAATGAGGAATCGTCGTTACATTTTTAGGAAAGGTCCATTGATTGTTTATGGAACTAAA
AlRPL4C     598    AAAATGAGGAATCGTCCATATGTTTCGCGTAAAGGTCCGTTGATTGTGTATGGAAATGAG
EsRPL4C     598    AAAATGAGGAATCGTAGATACATTTCTCGTAAAGGTCCTTTGATTGTTTACGGAAACGAA
```

```
RPL4D        661   GGAGCCAAGATTGTGAAAGCTTTTAGGAATCTTCCTGGTGTTGAGCTTTGTCACGTTGAG
AlRPL4B      361   GGATC   GTTAGTGGAAGCTTTTAGGAACATTACAGGGATTGATATATGTAATGTAGAG
AlRPL4C      658   GGAGCTAAGTTGGTTAAGGCGTTTAGGAACATTCCTGGGATTGATTTGTGTCATGTAGAG
CagraRPL4C   412                     GCGTTTAGGAACATTCTTGGGATTGATTTGTGTCATGTAGAG
EsRPL4C      658   GGAGCTAAGTTAGTGAAAGCGTTTAGGAACATTCCTGGGATTGATTTGTGTCATGTTGAG

RPL4D        721   AGGCTTAACTTGTTGAAATTAGCCCCTGGTGGTCACCTTGGTAGGTTTGTGATTTGGACT
AlRPL4B      418   AGATTGAGTCTATTGAAGTTAGCTCCTGGTGGTCATCTTGGTAGGTTTGTGATTTGGACT
AlRPL4C      718   AGGTTGAGTTTGCTTAAATTAGCTCCTGGTGGTCATTTAGGTAGATTTGTTGTGTGGACT
CagraRPL4C   454   AGGTTGAGTTTGCTTAAGTTAGCTCCTGGTGGTCACTTGGGGAGATTTGTGGTGTGGACT
EsRPL4C      718   AGGTTGAGTTTGTTAAAATTGGCTCCTGGTGGTCACTTGGGAAGATTCGTTGTGTGGACT

RPL4D        781   AAGTCTGCTTTTGAGAAGCTTGAATCTATCTATGGCTCGTTTGAGAAACCATCAGAGAAG
AlRPL4B      478   AAGTCTGCATTTGAGAAGCTTGAGTCTATTTACGGCTCGTTTGAGAAGTTATC
AlRPL4C      778   AAATCGGCTTTTGCGAAGTTGGAATTGGTTTATGGTTCGTTTGAGATGTCTTCGGAGATG
CagraRPL4C   514   AAATCGGCTTTCGAGAAGTTGGAATCGGTTTATGGTTCGTTTGAGAGGCCGGCAGAGATG
EsRPL4C      778   AAATCGGCTTTTGAGAAGTTGGAATCGGTTTACGGATCGTTTGAGAAGCCATCGGAGATG

RPL4D        841   AAGAAGGGTTACGTCTTGCCTCGTGCGAAGATGGTGAATGCTGATCTTGCTAGGATTATT
AlRPL4B      531       AAGGATATGTTTTGCTGCGGCCGAAGATGTTGAATGCTGATCTTGCTAGGATTATA
AlRPL4C      838   AAGAAAGGTTATGTTTTACCTAGGGCGAAGATGGTTAATGCTGATTTGGCTAGGATTATT
CagraRPL4C   574   AAGAGTGGTTATGTTTTACC
RPL4C        2                                      ATGAATGCTGATTTGGCTAGGATTATT
EsRPL4C      838   AAGAAGGGTTATGTTTTGCCTCGTCCGAAGATGGTGAATGCTGACCTTGCTAGGATTATC

RPL4D        901   AACTCCGATGAGGTACAGAGTGTGGTGAACCCGATTAAGGATGGTTCCAAGAGAGCGGTT
AlRPL4B      587   AACTCTGATGAGGTTTAGAGTGTGGTGAAGCCAATGAAGAAGGATGTGAAAAGAGGTGTG
AlRPL4C      898   AATTCGGATGAGGTTCAGAGTGTTGTTAGACCGATTGA-GATGGCGTGAAGAGAGCTGTG
CagraRPL4C   595                                                      AGAGCTGTG
RPL4B        1                                      ATGAAGAAGGATGTGAAGAGAGGTGTG
RPL4C        28    AATTCTGATGAGGTTCAGAGTGTGGTTAGACCGATTGAGAAAGATGTGAAGAGTGCTGTG
EsRPL4C      898   AATTCTGATGAGGTTCAGAGTGTGGTTAGGCCGATTAAGAAGGATGCTAAGAGAGCTGTG

RPL4D        961   CTGAAGAAGAATCCATTGAAGAACCTTAATGTGATGTTCAAGTTGAATCCTTATGCTAAG
AlRPL4B      647   ATTATGAAGAATCCTTTGAAGAATTTGAATGTGATGGTTAAGCTTAGTCATTACGCTAAG
AlRPL4C      958   TTGAAGAAGAATCCTCTTAAGAATTTGAATGTGATGTTGAAGCTTAACCCTTATGCGAAG
CagraRPL4C   604   TTGAAGAAGAATCCTCTTAAGAATTTGAATATGATGTTGAAGCTTAACCCTTACACTAAG
RPL4B        28    ATTATGAAGAATCCTTCGAATAATTTAAATTTGATGGTTAAGCTTAATCCTTATGCTAAG
RPL4C        88    TTGAAGAAGAATCCTCTTAAGAATTTGAATGTGATGTTGAAGCTTAACCCTTATGCTAAG
EsRPL4C      958   TTGAAGAAGAATCCTTTGAAGAACTTGAATGTGATGTTGAAGCTTAACCCTTATGCCAAG

RPL4D        1021  ACCGCAAAGAGAATGTCTCTGTTGGCTGAAGCTTCAAGGGTTAAGGCTAAGAAGGAGAAG
AlRPL4B      707   ACCGCGAAGATGATGTCTTTGTTGGCTGAAGCTCAGAGAGTGAAAGCTAAGAAATAGAAG
AlRPL4C      1018  ACTGCGAGGAGAATGTCTTTGTTGGCTGAAGCTGAGAGAGTGAAGTCGAAAAACGAGAAG
CagraRPL4C   664   ACTGCGAGGAGAATGTCTTTGTTGGCTGAAGCTGAGAGAGTAAAGTCCAAAAAGGAGAAG
RPL4B        88    ACCTCGAAGAGGATGTCATTATTGGCTGAAGCTCAGAGAGTGAAAGCTAAGAAGGAGAAG
RPL4C        148   ACTGTGAGGAGGATGTCTTTGTTGGCTGAAGCTCAGAGAGTTAAGTCGAAAAAGGAGAAG
EsRPL4C      1018  ACTGCGAGAAGGATGTCTCTTTTGGCTGAAGCTGAGAGGGTTAAGTCCAAAAAGGAGAAG

RPL4D        1081  CTCGAGAAGAAGAGGAAAGTCGTCACTAAGGAGGAGGCCCAAGCGATCAAAGCAGCAGGC
AlRPL4B      767   CTTGAGAAGAAGAGGAAACCCATTCCTAAGG
AlRPL4C      1078  CTTGAGAGGAAGAGAAAACCCATCTCAAAGGAAGGAGGCATGAAGATCAAAGCAGCAGGA
CagraRPL4C   724   CTCGAGAGGAAGAGGACACCTATCTCAAAGGAGGAAGCAGTGAAGATTAAAGCAGCAGGA
RPL4B        294   CTCGAGAGGAAGAGGAAA          AGGAGGAGGCAATGAAGATTAGATCAGCAGGG
RPL4C        208   CTCGAGAGGAAGAGGAAA                          GATCAAAGCAGCAGGA
EsRPL4C      1078  CTCGAGAGGAAGAGGAAA                          GATTAAAGCAGCAGGA

RPL4D        1141  AAGGCTTGGTATCAGACTATGATTTCAGACAGTGACTACACCGAGTTCGATAACTTCACC
AlRPL4B      957   AAGTCATGGTACAAGACCATGATCTCTGACAGTGACTACACCGAGTTTGACAACTTCTCT
AlRPL4C      1316  AAACCGTGGTACAGGACTATGATATCGGACAGTGACTACACTGAGTTTGACAACTTCACC
CagraRPL4C   784   AAAGCTTGTTACCAGACTATGATATCAGACAGTGACTACACAGAGTTTGATAACTTCACC
RPL4B        326   AAGTCATGGTACAAGACCATGATCTCGGACAGTGACTACCCCGAGTTTGACAACTTCTCT
RPL4C        453   AAAGCATGGTACCAGACTATGATATCAGATAGTGACTACACTGACTTTGATAACTTCACA
EsRPL4C      1305  AAAGCGTGGTACCAGACTATGATATCAGACAGTGACTACACCGAGTTTGATAACTTCACC

RPL4D        1201  AAGTGGCTTGGCGCTAGTCAGTAA  1224
AlRPL4B      1017  AAGTGGCTCGG               1027
AlRPL4C      1376  AAGTGGCTCGG               1386
CagraRPL4C   844   AAGTGGCTCGGCGTCGAACAGTAC  867
CrRPL4C
RPL4B        386   AAGTGGCTCGGTGTCGAACAGTAA  805
RPL4C        515   AAGTGGCTCGGTGTCGAACAGTAA  525
EsRPL4C      1365  AAGTGGCTTGGTGTGAATCAGTAA  1388
```

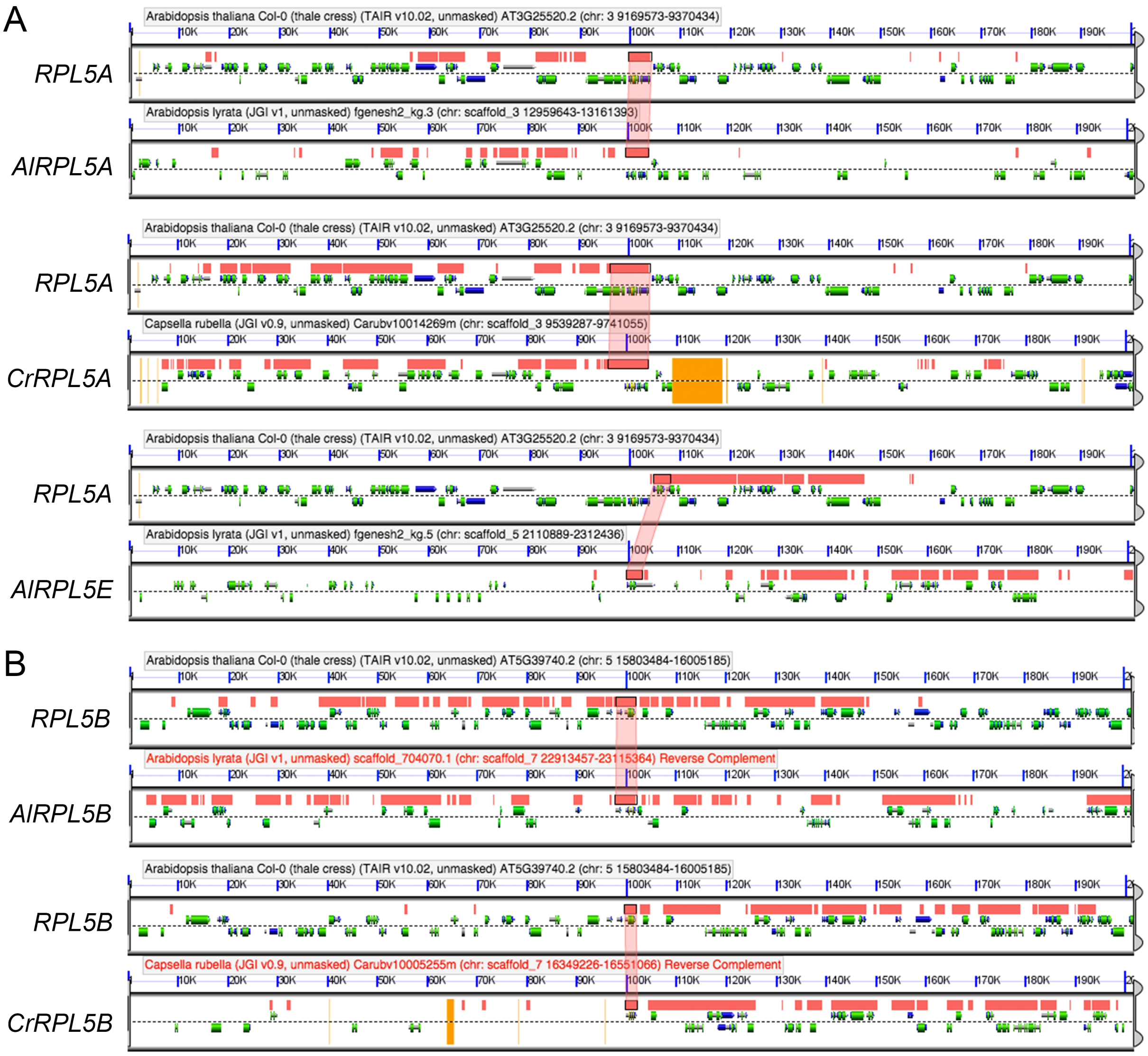

A
Arabidopsis thaliana Col-0 (thale cress) (TAIR v10.02, unmasked) AT3G25520.2 (chr: 3 9169573-9370434)
RPL5A
Arabidopsis lyrata (JGI v1, unmasked) fgenesh2_kg.3 (chr: scaffold_3 12959643-13161393)
AlRPL5A
Arabidopsis thaliana Col-0 (thale cress) (TAIR v10.02, unmasked) AT3G25520.2 (chr: 3 9169573-9370434)
RPL5A
Capsella rubella (JGI v0.9, unmasked) Carubv10014269m (chr: scaffold_3 9539287-9741055)
CrRPL5A
Arabidopsis thaliana Col-0 (thale cress) (TAIR v10.02, unmasked) AT3G25520.2 (chr: 3 9169573-9370434)
RPL5A
Arabidopsis lyrata (JGI v1, unmasked) fgenesh2_kg.5 (chr: scaffold_5 2110889-2312436)
AlRPL5E
B
Arabidopsis thaliana Col-0 (thale cress) (TAIR v10.02, unmasked) AT5G39740.2 (chr: 5 15803484-16005185)
RPL5B
Arabidopsis lyrata (JGI v1, unmasked) scaffold_704070.1 (chr: scaffold_7 22913457-23115364) Reverse Complement
AlRPL5B
Arabidopsis thaliana Col-0 (thale cress) (TAIR v10.02, unmasked) AT5G39740.2 (chr: 5 15803484-16005185)
RPL5B
Capsella rubella (JGI v0.9, unmasked) Carubv10005255m (chr: scaffold_7 16349226-16551066) Reverse Complement
CrRPL5B

Figure S5

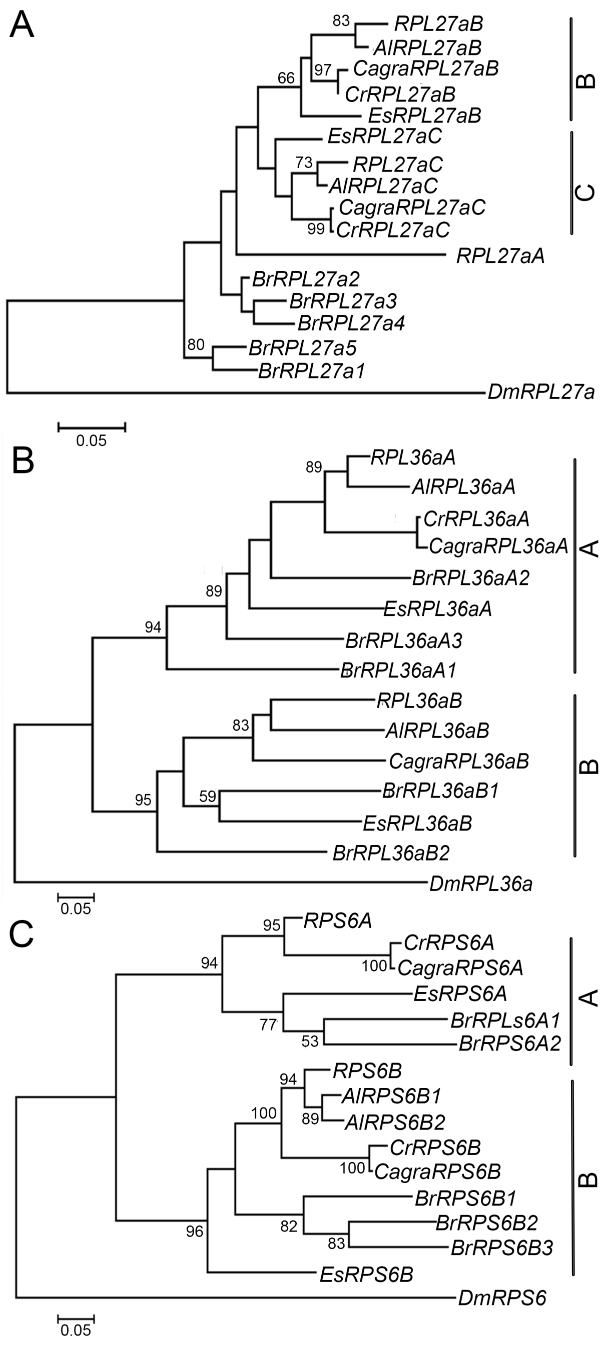
A
RPL27aB
AlRPL27aB
CagraRPL27aB
CrRPL27aB
EsRPL27aB
EsRPL27aC
RPL27aC
AlRPL27aC
CagraRPL27aC
CrRPL27aC
RPL27aA
BrRPL27a2
BrRPL27a3
BrRPL27a4
BrRPL27a5
BrRPL27a1
DmRPL27a
83
97
66
73
99
80
B
C
0.05
B
RPL36aA
AlRPL36aA
CrRPL36aA
CagraRPL36aA
BrRPL36aA2
EsRPL36aA
BrRPL36aA3
BrRPL36aA1
RPL36aB
AlRPL36aB
CagraRPL36aB
BrRPL36aB1
EsRPL36aB
BrRPL36aB2
DmRPL36a
89
89
94
83
59
95
A
B
0.05
C
RPS6A
CrRPS6A
CagraRPS6A
EsRPS6A
BrRPLs6A1
BrRPS6A2
RPS6B
AlRPS6B1
AlRPS6B2
CrRPS6B
CagraRPS6B
BrRPS6B1
BrRPS6B2
BrRPS6B3
EsRPS6B
DmRPS6
95
94
100
77
53
94
89
100
100
82
83
96
A
B
0.05

Figure S6

Table S1. *RPL9* genes in Brassicaceae species

Organism	Name in phylogeny	Phytozome ID	Chromosome /Scaffold	Amino acids	Notes
Arabidopsis thaliana	*RPL9B*	AT1G33120	Chr 1	194	*RPL9B* and *RPL9C* are separated by approximately 10.7kb
	RPL9C	AT1G33140	Chr1	194	
	RPL9D	AT4G10450	Chr 4	194	
Arabidopsis lyrata	*AlRPL9B*	473492	Scaffold 1 gene 1	194	*AlRPL9B* and *AlRPL9C* are separated by approximately 8.1kb
	AlRPL9C	473492	Scaffold 1 gene 2	194	
	AlRPL9D	942855	Scaffold 6	194	
Capsella rubella	*CrRPL9B*	Carubv10001828	Scaffold 6	194	
	CrRPL9C	scaffold_1:11,674,298..11,676,297	Scaffold 1	194	
Capsella grandiflora	*CagraRPL9B*	Cagra.14151s0004	Scaffold14151	194	
	CagraRPL9C1	Cagra.18493s0001	Scaffold18493	194	CagraRPL9C1 and CagraRPL9C2 are separated by 38bp
	CagraRPL9C2	Cagra.18493s0001	Scaffold18493	194	
Brassica rapa	*BrRPL9B*	Bra035980	Scaffold 000111	194	
	BrRPL9C	Bra039999	Scaffold 000185	194	
	BrRPL9D	Bra033171	Ch A02	194	
	BrRPL9E	Bra037917	ChA09	194	
Eutrema salsugineum	*EsRPL9B*	Thhalv10008838m	Scaffold 5 gene 1	194	*EsRPL9D* and *EsRPL9E* are separated by approximately 12.1kb
	EsRPL9C	Thhalv10008851m	Scaffold 5 gene 2	194	
	EsRPL9D	Thhalv10029481	Scaffold 3 gene 1	194	*EsRPL9D* and *EsRPL9E* are separated by approximately 9.1kb
	EsRPL9E	Thhalv10028984m	Scaffold 3 gene 2	190	

Table S2. *RPL9* genes in eudicot species

Organism	Gene name	Phytozome ID	Chromosome /Scaffold	Amino acids	Notes
Linum usitatissimum	*LuRPL9A*	Lus10005286	Scaffold 109	194	
	LuRPL9B	Lus10032962	Scaffold 51	194	
	LuRPL9C	Lus10015588	Scaffold 233	194	
	LuRPL9D	Lus10032918	Scaffold 51	194	
Populus trichocarpa	*PtRPL9A*	Potri.001G453900.1	Chr 1	193	*PtRPL9A* and *PtRPL9B* are separated by approximately 4kb
	PtRPL9B	Potri.001G454000.1	Chr 1	193	
	PtRPL9C	Potri.011G147700.1	Chr 11	193	*PtRPL9C* and *PtRPL9D* are separated by approximately 40kb
	PtRPL9D	Potri.011G148700.2	Chr 11	193	
Medicago truncatula	*MtRPL9A*	Medtr3g093110.1	Chr 3	193	
	MtRPL9B	Medtr5g068780.1	Chr 5	192	
Phaseolus vulgaris	*PvRPL9A*	Phvul.004G046700.1	Chr 4	193	
	PhvRPL9B	Phvul.007G076200.1	Chr 7	192	
	PhvRPL9C	Phvul.009G039400.1	Chr 9	193	*PvRPL9C* and *PvRPL9D* are separated by approximately 10kb
	PhvRPL9D	Phvul.009G039600.1	Chr 9	193	
Carica papaya	*CpRPL9A*	evm.model.supercontig183.14		194	*CpRPL9A* and *CpRPL9B* are separated by approximately 1kb
	CpRPL9B	evm.model.supercontig183.15		194	
	CpRPL9C	evm.model.supercontig27.99		194	
Gossypium raimondii	*GrRPL9A*	Gorai.003G072800.2	Chr 3	194	
	GrRPL9B	Gorai.004G127700.1	Chr 4	194	
	GrRPL9C	Gorai.007G018400.1	Chr 7	194	
	GrRPL9D	Gorai.008G262000.1	Chr 8	194	
Citrus sinensis	*CsRPL9A*	orange1.1g029362	Scaffold 00230	194	
	CsRPL9B	orange1.1g029415	Scaffold 00009	194	
	CsRPL9C	orange1.1g029373	Scaffold 00027	194	
Citrus clementina	*CcRPL9A*	Ciclev10009377	Scaffold 1	194	
	CcRPL9B	Ciclev10022376	Scaffold 3	194	
	CcRPL9C	Ciclev10022372	Scaffold 3	194	
Vitis vinifera	*VvRPL9A*	GSVIVG01016795001	Chr 9	193	
	VvRPL9B	GSVIVG01038670001	Chr 16	193	
Solanum tuberosum	*StRPL9A*	PGSC0003DMP400046713	Chr 6	187	
	StRPL9B	PGSC0003DMT400064920	Chr 8	194	
	StRPL9C	PGSC0003DMP400050946	Chr 12	194	
Solanum lycopersicum	*SlRPL9A*	Solyc06g073310.2.	Chr 6	194	
	SlRPL9B	Solyc08g014550.2.1	Chr 8	194	
	SlRPL9C	Solyc12g096700.1.1	Chr 12	194	
Mimulus guttatus	*MgRPL9A*	mgv1a014306	Scaffold 8	194	
	MgRPL9B	mgv1a014281	Scaffold 104	194	
Aquilegia coerulea	*AcRPL9A*	Aquca_037_00307.1	Scaffold 37	194	
	AcRPL9B	Aquca_095_00020.1	Scaffold 95	194	

Table S3. *RPL9* genes in monocot species

Organism	Name in phylogeny	Phytozome ID	Chromosome /Scaffold	Amino acids	Notes
Sorghum bicolor	*SbRPL9A*	Sobic.004g003000	Chr 4	125	
	SbRPL9B	Sobic.010G005300	Chr 10	190	
Zea mays	*ZmRPL9A*	GRMZM2G167103	Chr 4	190	
	ZmRPL9B	GRMZM2G385287	Chr 6	190	
	ZmRPL9C	GRMZM2G084739	Chr 9	190	
Setaria italica	*SiRPL9A*	Si018430m	Scaffold 1	190	
	SiRPL9B	Si007334m	Scaffold 4	189	
Panicum virgatum	*PvRPL9A*	Pavir.Aa03564	Chr 01a	190	
	PvRPL9B	Pavir.Ab00063	Chr 01b	190	
	PvRPL9C	Pavir.J21269	contig231585	190	
	PvRPL9D	Pavir.J39022.1	contig80767	190	
Oryza sativa	*OsRPL9A*	LOC_Os02g01332.1	Chr 2	192	
	OsRPL9B	LOC_Os09g31180.1	Chr 9	190	
Brachypodium distachyon	*BdRPL9A*	Bradi1g21850.1	Chr 1	189	
	BdRPL9B	Bradi1g52040.1	Chr 1	189	
	BdRPL9C	Bradi3g00640.1	Chr 3	189	

Table S4. *RPL4* genes in Brassicaceae

Organism	Name in phylogeny	Phytozome ID	Chromosome/ Scaffold	Amino acids	Notes
Arabidopsis thaliana	*RPL4A*	AT3G09630	Ch3	406	
	RPL4B	AT1G35200	Ch1	N.A.	Pseudogene
	RPL4C	AT2G24730	Ch 2	N.A.	Pseudogene
	RPL4D	AT5G02870	Ch 5	407	
Arabidopsis lyrata	*AlRPL4A*	478282	Scaffold 3	406	
	AlRPL4B	314007	Scaffold 1	243	Pseudogene
	AlRPL4C	320281	Scaffold 4	394	Pseudogene
	AlRPL4D	487065	Scaffold 6	407	
Capsella rubella	*CrRPL4A*	Carubv10013852m	Scaffold 3	406	
	CrRPL4C	Carubv10025452m	Scaffold 4	172	Pseudogene
	CrRPL4D	Carubv10001096m	Scaffold 6	407	
Capsella grandiflora	*CagraRPL4A*	Cagra.2515s0053	Scaffold2515	406	
	CagraRPL4C	Cagra.15158s0002	Scaffold 15158	197	Pseudogene
	CagraRPL4D	Cagra.2240s0018	Scaffold2240	407	
Brassica rapa	*BrRPL4A1*	Brara.A03522	Ch A01	406	
	BrRPL4A2	Brara.E03076	Ch A05	406	
	BrRPL4D1	Brara.C00088	Ch A03	406	
	BrRPL4D2	Brara.J02849	Ch A010	406	
Eutrema salsugineum	*EsRPL4A*	Thhalv10020851m	Scaffold 13	406	
	EsRPL4C	Thhalv10000184m	Scaffold 15	406	
	EsRPL4D	Thhalv10013700m	Scaffold 2	406	

Table S5. *RPL5* genes in Brassicaceae

Organism	Name in phylogeny	Phytozome ID	Chromosome /Scaffold	Amino acids	Notes
Arabidopsis thaliana	*RPL5A*	AT3G25520	Ch 3	301	
	RPL5B	AT5G39740	Ch 5	301	
	RPL5C	AT5G40130	Ch 5	120	*RPL5B* and *RPL5C* are separated by approximately 150kb
Arabidopsis lyrata	*AlRPL5A*	480054	Scaffold 3	301	
	AlRPL5B	947841	Scaffold 7	302	
	AlRPL5E	898932	Scaffold 5	301	
Capsella rubella	*CrRPL5A*	Carubv10014269m	Scaffold 3	301	
	CrRPL5B	Carubv10005357m	Scaffold 7	301	
Capsella grandiflora	*CagraRPL5A*	Cagra.1183s0004	Scaffold1183	301	
	CagraRPL5B	Cagra.6211s0001	Scaffold6211	301	
Brassica rapa	*BrRPL5D1*	Brara.C04002	Ch A03	302	
	BrRPL5D2	Brara.G00424	Ch A07	302	
	BrRPL5E	Brara.F03014	Ch A06	303	
Eutrema salsugineum	*EsRPL5D*	Thhalv10002621m	Scaffold 4	301	
	EsRPL5E	Thhalv10004670m	Scaffold 6	303	

Table S6. *RPL27a* genes in Brassicaceae

Organism	Name in phylogeny	Phytozome ID	Chromosome /Scaffold	Amino acids	Notes
Arabidopsis thaliana	*RPL27aA*	AT1G12960	Ch 1	104	Pseudogene
	RPL27aB	AT1G23290	Ch 1	146	
	RPL27aC	AT1G70600	Ch 1	146	
Arabidopsis lyrata	*AlRPL27aB*	472587	Scaffold 1	146	
	AlRPL27aC	476190	Scaffold 2	146	
Capsella rubella	*CrRPL27aB*	Carubv10011206m	Scaffold 1	146	
	CrRPL27aC	Carubv10021115m	Scaffold 2	146	
Capsella grandiflora	*CagraRPL27aB*	Cagra.0605s0079	Scaffold605	146	
	CagraRPL27aC	Cagra.1777s0010	Scaffold1777	146	
Brassica rapa	*BrRPL27a1*	Brara.B01921	Ch A02	146	
	BrRPL27a2	Brara.G02437	Ch A07	146	
	BrRPL27a3	Brara.G00984	Ch A07	146	
	BrRPL27a4	Brara.H02216	Ch A08	147	
	BrRPL27a5	Brara.I03248	Ch A09	146	
Eutrema salsugineum	*EsRPL27aB*	Thhalv10009027m	Scaffold 5	146	
	EsRPL27aC	Thhalv10019240m	Scaffold 9	146	

Table S7. *RPL36a* genes in Brassicaceae

Organism	Name in phylogeny	Phytozome ID	Chromosome/Scaffold	Amino acids	Notes
Arabidopsis thaliana	*RPL36aA*	AT3G23390	Ch 3	105	
	RPL36aB	AT4G14320	Ch 4	105	
Arabidopsis lyrata	*AlRPL36aA*	479854	Scaffold 3	105	
	AlRPL36aB	946959	Scaffold 7	105	
Capsella rubella	*CrRPL36aA*	Carubv10014940m	Scaffold 3	105	
	CrRPL36aB	Carubv10005895	Scaffold 7	105	
Capsella grandiflora	*CagraRPL36aA*	Cagra.2181s0002	Scaffold2181	105	
	CagraRPL36aB	Cagra.1618s0003	Scaffold1618	105	
Brassica rapa	*BrRPL36aA1*	Brara.A02648	Ch A01	105	
	BrRPL36aA2	Brara.C03915	Ch A03	105	
	BrRPL36aA3	Brara.G00538	Ch A07	105	
	BrRPL36aB1	Brara.D00648	Ch A04	105	
	BrRPL36aB2	Brara.H01878	Ch A08	105	
Eutrema salsugineum	*EsRPL36aA*	Thhalv10021815m	Scaffold 13	105	
	EsRPL36aB	Thhalv10026643m	Scaffold 1	105	

Table S8. *RPL6* genes in Brassicaceae

Organism	Name in phylogeny	Phytozome ID	Chromosome /Scaffold	Amino acids	Notes
Arabidopsis thaliana	*RPS6A*	AT4G31700	Ch 4	250	
	RPS6B	AT5G10360	Ch 5	249	
Arabidopsis lyrata	*AlRPS6B1*	487866	Scaffold 6	249	
	AlRPS6B2	915134	Scaffold 9	249	
Capsella rubella	*CrRPS6A*	Carubv10005584m	Scaffold 7	250	
	CrRPS6B	Carubv10001803m	Scaffold 6	249	
Capsella grandiflora	*CagraRPS6A*	Cagra.3807s0038	Scaffold3807	198	
	CagraRPS6B	Cagra.1085s0023	Scaffold1085	249	
Brassica rapa	*BrRPS6A1*	Brara.A00605	Ch A01	249	
	BrRPS6A2	Brara.H01340	Ch A08	250	
	BrRPS6B1	Brara.C00414	Ch A03	249	
	BrRPS6B2	Brara.B00353	Ch A02	250	
	BrRPS6B3	Brara.J02318	Ch A010	249	
Eutrema salsugineum	*EsRPS6A*	Thhalv10026073m	Scaffold 1	250	
	EsRPS6B	Thhalv10014497m	Scaffold 2	249	
